# Structural basis of allosteric and bitopic ligands binding in sphingosine-1-phosphate receptors 2 and 3

**DOI:** 10.1093/procel/pwaf068

**Published:** 2025-08-20

**Authors:** Yanhong Wu, Qiuru Chen, Hongyu Wang, Kezhen Liu, Jiaxin Wei, Mu Wang, Kun Chen, Ya Zhu, Shuo Han, Cuiying Yi, Limin Ma, Gisela Schnapp, Alexander Pautsch, Christian Gnamm, Matthias Grauert, Esther Schmidt, Qiuxiang Tan, Beili Wu, Qiang Zhao

**Affiliations:** State Key Laboratory of Drug Research and State Key Laboratory of Chemistry Biology, Shanghai Institute of Materia Medica, Chinese Academy of Sciences, Shanghai 201203, China; University of Chinese Academy of Sciences, Beijing 100864, China; School of Life Science and Technology, ShanghaiTech University, Shanghai 201210, China; State Key Laboratory of Drug Research and State Key Laboratory of Chemistry Biology, Shanghai Institute of Materia Medica, Chinese Academy of Sciences, Shanghai 201203, China; School of Life Science and Technology, ShanghaiTech University, Shanghai 201210, China; State Key Laboratory of Drug Research and State Key Laboratory of Chemistry Biology, Shanghai Institute of Materia Medica, Chinese Academy of Sciences, Shanghai 201203, China; University of Chinese Academy of Sciences, Beijing 100864, China; State Key Laboratory of Drug Research and State Key Laboratory of Chemistry Biology, Shanghai Institute of Materia Medica, Chinese Academy of Sciences, Shanghai 201203, China; University of Chinese Academy of Sciences, Beijing 100864, China; State Key Laboratory of Drug Research and State Key Laboratory of Chemistry Biology, Shanghai Institute of Materia Medica, Chinese Academy of Sciences, Shanghai 201203, China; University of Chinese Academy of Sciences, Beijing 100864, China; State Key Laboratory of Drug Research and State Key Laboratory of Chemistry Biology, Shanghai Institute of Materia Medica, Chinese Academy of Sciences, Shanghai 201203, China; School of Life Science and Technology, ShanghaiTech University, Shanghai 201210, China; State Key Laboratory of Drug Research and State Key Laboratory of Chemistry Biology, Shanghai Institute of Materia Medica, Chinese Academy of Sciences, Shanghai 201203, China; University of Chinese Academy of Sciences, Beijing 100864, China; Lingang Laboratory, Shanghai 200031, China; State Key Laboratory of Drug Research and State Key Laboratory of Chemistry Biology, Shanghai Institute of Materia Medica, Chinese Academy of Sciences, Shanghai 201203, China; State Key Laboratory of Drug Research and State Key Laboratory of Chemistry Biology, Shanghai Institute of Materia Medica, Chinese Academy of Sciences, Shanghai 201203, China; State Key Laboratory of Drug Research and State Key Laboratory of Chemistry Biology, Shanghai Institute of Materia Medica, Chinese Academy of Sciences, Shanghai 201203, China; Boehringer-Ingelheim Pharma GmbH & Co. KG, Department of Medicinal Chemistry, Biberach 88397, Germany; Boehringer-Ingelheim Pharma GmbH & Co. KG, Department of Medicinal Chemistry, Biberach 88397, Germany; Boehringer-Ingelheim Pharma GmbH & Co. KG, Department of Medicinal Chemistry, Biberach 88397, Germany; Boehringer-Ingelheim Pharma GmbH & Co. KG, Department of Medicinal Chemistry, Biberach 88397, Germany; Boehringer-Ingelheim Pharma GmbH & Co. KG, Department of Drug Discovery Sciences, Biberach 88397, Germany; State Key Laboratory of Drug Research and State Key Laboratory of Chemistry Biology, Shanghai Institute of Materia Medica, Chinese Academy of Sciences, Shanghai 201203, China; State Key Laboratory of Drug Research and State Key Laboratory of Chemistry Biology, Shanghai Institute of Materia Medica, Chinese Academy of Sciences, Shanghai 201203, China; University of Chinese Academy of Sciences, Beijing 100864, China; School of Pharmaceutical Science and Technology, Hangzhou Institute for Advanced Study, UCAS, Hangzhou 310024, China; School of Life Science and Technology, ShanghaiTech University, Shanghai 201210, China; State Key Laboratory of Drug Research and State Key Laboratory of Chemistry Biology, Shanghai Institute of Materia Medica, Chinese Academy of Sciences, Shanghai 201203, China; University of Chinese Academy of Sciences, Beijing 100864, China; Zhongshan Institute of Drug Discovery, SIMM, CAS, Zhongshan 528400, China

## Dear Editor,

Allosteric ligands of G protein-coupled receptors offer advantages over orthosteric ligand in subtype selectivity, spatiotemporal sensitivity, and potentially biased signaling, yet their discovery remains challenging. Here, we report two crystal structures of human sphingosine-1-phosphate receptor 3 (S1P3) in complex with the simultaneously binding of bitopic ligand SPM-242 and allosteric ligands Cpd-32 or CYM52581. To further reveal the inhibition mechanism of antagonists and ligand subtype selectivity, two cryo-electron microscopy structures of S1P_2_ and S1P_3_ in complex with heterotrimeric G_i_ protein were solved. The S1P_3_ complexes reveal an allosteric site that lies outside of the helical bundle in S1P_3_ receptor, which is a new site recognized by allosteric modulators in class A GPCR. Structural comparison further explains the selectivity of bitopic antagonist SPM-242 and allosteric antagonists CYM52581 on S1P_3_ over S1P_1_. These structural studies, together with functional assays, provide structural insights into the bitopic and allosteric antagonism of class A GPCRs, which will further facilitate the design of selective drugs targeting these receptors.

**Figure 1.**
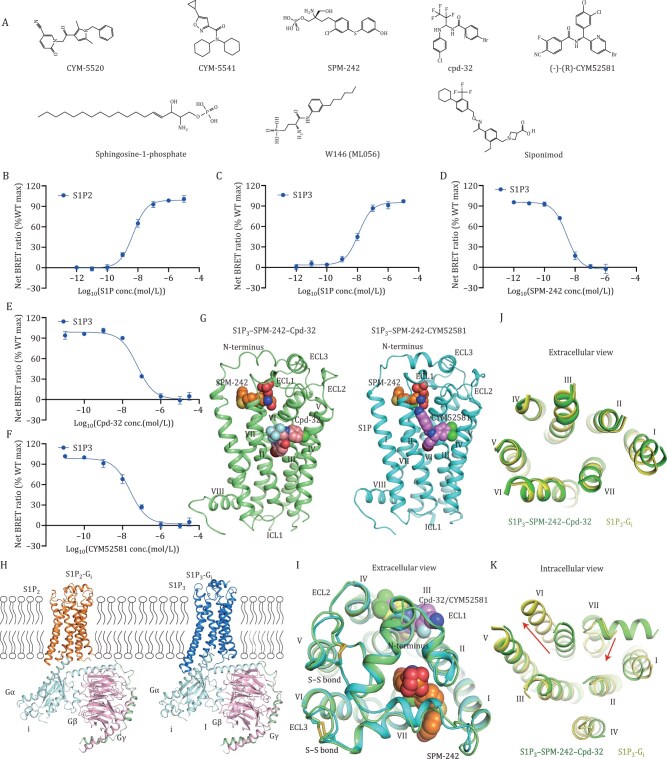
**Structural, functional, and pharmacological profiling of S1P receptors modulators.** (A) Structure of different ligands: CYM-5520, CYM-5541, (R)-SPM-242, Cpd-32, CYM52581, S1P, W146, and Siponimod. (B and C) TRUPATH assays of agonist S1P induced G_i_ accumulation of wild-type S1P_2_ (B) and S1P_3_ (C). (D–F) Trupath assay of antagonist SPM-242 (D), Cpd-32 (E), and (CYM52581) (F) induced G_i_ accumulation of wild-type S1P_3_. At least three independent experiments were performed in duplicate, and data are shown as mean ± SEM. Supplementary Tables S3 and S4 for detailed statistical evaluation. (G) Crystal structures of S1P_3_ with SPM-242 and Cpd-32, and S1P_3_ with SPM-242 and CYM52581. The structures are shown in cartoon representation, with the receptors colored lime and cyan, respectively. The ligands SPM-242, Cpd-32 and CYM52581 are shown in spheres representation and colored orange, yellow, and violet, respectively. (H) Cryo-EM structures of S1P_2_–G_i_ and S1P_3_–G_i_ complexes. S1P_2_ and S1P_3_ are shown in orange and marine cartoon representation, respectively. Gα_i_, Gβ, and Gγ are colored light-cyan, light-pink, and light-green, receptively. (I) Extracellular view of crystal structures. The disulfide bonds are shown as yellow sticks. (J and K) Extracellular view (J) and Intracellular view (K) of inactive and active S1P3 structures. The red arrows indicate the large conformation change of helix VI and VII.

The S1P receptor family (S1P_1_–S1P_5_) regulates cell survival, cell migration, and inflammation. Endogenous agonist Sphingosine-1-phosphate (S1P), derived from the sphingomyelin metabolism, is abundant (> 200 nmol/L) in plasma (Hammad et al., 2012). Among the S1Ps, S1P_2_ suppresses tumor angiogenesis bone loss, while S1P_3_ drives metastasis and disrupts the blood-tumor barrier, making it attractive therapeutic target for cardioprotection, fibrosis-related diseases, and breast cancer. However, existing ligands (e.g. JTE-013) (Chew et al., 2016) lack specificity or potency, hindering clinical translation (Fig. 1A). Thus, allosteric agonists, as well as the novel bitopic agonist for S1P_2_ and S1P_3_ had been screened and CYM-5520 and CYM-5541, which had been used as the therapy for osteoporosis and ischemia-reperfusion injury, were discovered (Jo et al., 2012; Satsu et al., 2013) (Fig. 1A–C). In addition, SPM-242 was screened for treating multiple diseases, including sepsis and asthma (Jo et al., 2012; Yasushi Kohno et al., 2004) (Fig. 1A and D). Meanwhile, Cpd-32 and CYM52581 have been investigated to treat pain and cardiopulmonary diseases, respectively (Fig. 1A, 1E and 1F).

Here, we present two crystal structures S1P3-SPM-242-Cpd-32 and S1P3-SPM-242-CYM52581 at 3.0 Å and 3.6 Å resolution, respectively (Fig. 1G, and Supplementary Table S1). To reveal its antagonism, we also report cryo-electron microscopy (cryo-EM) structures of S1P_2_ or S1P_3_ in complex with G_i_ protein (Fig. 1H). Among these four structures, S1P3-SPM-242-Cpd-32 and S1P3-SPM-242-CYM52581 exemplify the inactive states, while S1P2-Gi and S1P3-Gi complexes represent the active state of S1P2/S1P3 receptor (Supplementary Table S1). These engineered receptors retain native like binding of S1P, SPM-242, Cpd-32, and CYM52581 as well as synergistic stabilization of orthosteric and allosteric ligands (Supplementary Fig. S1A-D). Together with extensive functional assays, these structures provide a structural framework for bitopic and allosteric ligand-binding mode and selectivity as well as synergistic inhibition mechanisms of different types of antagonists (Supplementary Fig. S2A and S2B). Strong and unambiguous electron densities are observed for all ligands and their interacting residues in the S1P2 and S1P3 structures (Supplementary Fig. S3–S5).

The S1P3-SPM-242-Cpd-32 and S1P3-SPM-242-CYM52581 complexes share similar the seven-transmembrane (7TM) helical bundle with a Cα atom root-mean square-deviation (r.m.s.d.) of 0.61 Å (Fig. 1I). Thus, we will primarily focus on the S1P_3_–SPM-242–Cpd-32 complex due to its higher resolution. S1P_3_ adopts a canonical seven-transmembrane architecture, with its N-terminal α-helical occluding the extracellular ligand-binding pocket. Two disulfide bonds are observed within extracellular loop 2 (ECL2) and extracellular loop 3 (ECL3) of S1P_3_, which are coincident with S1P_1_, S1P_2_, and S1P_5_ structures (Chen et al., 2022; Yuan et al., 2021; Zhao et al., 2022) (Fig. 1I). The conserved disulfide bonds constrain the conformation of ECL2 and ECL3, further stabilizing the conformations of ECL1 and N terminus and enclosing the hydrophobic ligands (Fig. 1I). A structural comparison of the S1P3-SPM-242-Cpd-32 complex with the S1P_3_-G_i_ structure reveals similarities on the extracellular side (Fig. 1J). However, a significant conformational difference emerges in the transmembrane region: helix VI exhibits a pronounced outward displacement of 8.5Å (measured at the Cα of R237^6.29^), while helix VII shifts inward by 6.5 Å (measured by the Cα of T299^7.54^) (Fig. 1K). These movements highlight distinct rearrangement associated with S1P3 receptor activation.

SPM-242 contains a polar, zwitterionic head group and a hydrophobic tail (Jo et al., 2012) (Fig. 1A). It occupies both the orthosteric pocket shaped by N-terminus, helices Ⅱ-Ⅲ, ECL1, and an allosteric pocket formed by helices Ⅰ, Ⅱ, and Ⅶ, which is different from W146 (ML056), S1P and Siponimod in all solved S1P receptors (Chen et al., 2022; Liu et al., 2013; Hanson et al., 2012; Sanna et al., 2006; Yuan et al., 2021) (Fig. 1I, Supplementary Fig. S6Aand S6B). To accommodate SPM-242, the extracellular tip of helix Ⅰ in S1P_3_ strikes outwardly away from helix Ⅶ by 2 Å (referenced by Cα of T42) in comparison with the S1P_1_–W146 complex (Supplementary Fig. S6C and S6D). As a result, the binding site of SPM-242 is much closer to the extracellular surface when compared with W146, as it inserted in the gap between helices Ⅰ and Ⅶ instead of occupied in the helical bundle (Supplementary Fig. S6E).

**Figure 2.**
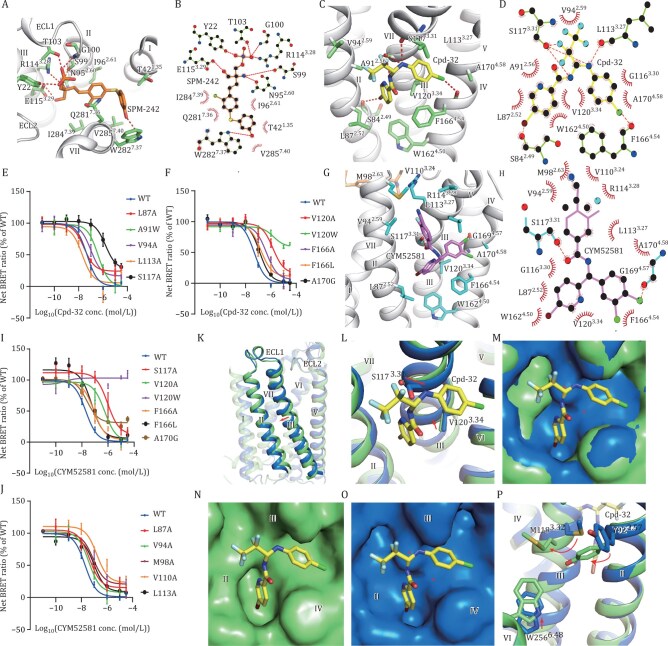
**Ligand-binding modes and interaction networks of S1P3 receptor.** (A) Ligand-binding pocket of SPM-242. S1P3 is shown in gray cartoon representation. The ligand SPM-242 (orange carbons) and S1P_3_ residues (lime carbons) are shown in sticks representation. Hydrogen bonds are red dashed lines. (B) LigPlot^+^ diagram indicates the S1P_3_ residues (lime, labeled according to Ballesteros-Weinsterin numbering) interacting with SPM-242 (orange carbons). (C) Ligand-binding pocket of Cpd-32. S1P_3_ is shown in gray cartoon representation. Cpd-32 (yellow carbons) and S1P_3_ residues (lime carbons) are shown as sticks. Hydrogen and halogen bonds are shown as red dashed lines. (D) LigPlot^+^ diagram indicates the S1P_3_ residues interact with Cpd-32 (yellow carbons). (E and F) Trupath assays of antagonist Cpd-32. At least three independent experiments were performed in duplicate, and data are shown as mean ± SEM. Supplementary Table S2 for detailed statistical evaluation. (G) Ligand-binding pocket of CYM52581. S1P_3_ is shown in gray cartoon representation. CYM52581 (violet carbons), SPM-242 (orange carbons), and S1P_3_ residues (cyan carbons) are shown as sticks. Hydrogen and halogen bonds are shown as red dashed lines. (H) Schematic representation of the interactions between S1P_3_ and CYM52581 analyzed using the LigPlot^+^ program. (I and J) Trupath assays of antagonist CYM52581. At least three independent experiments were performed in duplicate, and data are shown as mean ± SEM. Supplementary Table S2 for detailed statistical evaluation. (K) Structural superimposition of S1P_3_ in the inactive and active states. The inactive and active S1P_3_ structures are shown in cartoon representation, with the receptors colored lime and marine, respectively. Structural superimposition of inactive and active S1P_3_ shows relative movements in helix Ⅲ0020of those two structures, which are highlighted by a red arrow. (L) The conformational changes of allosteric ligand-binding site during receptor activation. S117^3.31^ and V120^3.34^ are shown as lime and marine sticks in the inactive and active S1P_3_, respectively. The red arrows indicate the conformational changes of those residues. Cpd-32 are shown as yellow sticks. (M–O) The structures of inactive and active S1P_3_ are shown in lime and marine surface representation, respectively. And allosteric ligand Cpd-32 are shown as yellow sticks. Cpd-32 binding surface on the inactive S1P_3_ (N) and the corresponding surface of active S1P_3_ with Cpd-32 (O). A red asterisk indicates the steric hinderance between Cpd-32 and the surface of active S1P_3_. (P) M118^3.32^, L122^3.36^ and W256^6.48^ in inactive and active S1P_3_ are shown as lime and marine sticks, respectively. Cpd-32 is shown in yellow sticks representation. The red arrows indicate the conformational changes of Y92^2.57^, M118^3.32^and W256^6.48^ in inactive S1P_3_ relative to those residues in the active S1P_3_.

SPM-242’s zwitterionic head mimics S1P’s phosphate/amine groups, mediating high-affinity ligand binding via polar interactions with Y22, N95^2.60^, R114^3.28^, E115^3.29^, S99^ECL1^, and T103^ECL1^ (superscript indicates residue number according to the Ballesteros-Weinstein nomenclature) (Ballesteros and Weinstein, Academic Press, 1995) (Fig. 2A and 2B). Alanine substitutions of Y22, N95^2.60^, R114^3.28^ or E115^3.29^ impaired S1P-induced G_q_ activation, while S99^ECL1^A /T103^ECL1^A mutations reduced SPM-242 inhibition by 10- and 22-fold, respectively (Supplementary Fig. S7A–C and Supplementary Table S2).

Unlike ligands such as S1P-bound W146 (Michael A. Hanson et al., 2012), the hydrophobic moiety of SPM-242 extends to a unique allosteric sites (helices Ⅰ, Ⅱ and Ⅶ) (Supplementary Fig. S6C and S6D). Non-conserved residue I284^7.39^ forms hydrophobic interactions with SPM-242’s chlorobenzene group, and its alanine mutation (7-fold binding loss) or leucine (complete inhibition loss) highlights the importance of steric constrains (Figs. 2A, 2B, Supplementary Fig. S7D, S7E and Supplementary Table S2). Upon binding of SPM-242, the large side chain of F46^1.39^ rotates about 70°C and forms hydrophobic interactions with I96^2.61^ and Y92^2.57^ in the helix II (Supplementary Fig. S6D). The side chain rotation pushes helix I outwardly to facilitate SPM-242 binding (Supplementary Fig. S6D). Replacement F46^1.39^ with alanine antagonistic effect by 2-fold, implying the importance of the interactions between F46^1.39^ (Supplementary Fig. S7F and Supplementary Table S2). Further, mutating T42^1.35^ and V285^7.40^ or W282^7.37^ moderately decreased the antagonistic effects of SPM-242 (Supplementary Fig. S7G–I and Supplementary Table S2). While Q281^7.36^ enhances the antagonist by disrupting unfavorable hydrophilic interactions (Supplementary Fig. S7J and Supplementary Table S2).

Cpd-32 and CYM52581, two structurally distinct S1P3 allosteric antagonists, share a lipid-facing pocket shaped by helices Ⅱ-IV (Fig. 1A and Supplementary Fig. S8A–C). Unlike PAR2 (AZ3451) and CB1 (ORG27569), which utilize helices II-IV as binding sites, the binding sites of Cpd-32 and CYM52581 are much closer towards the extracellular membrane surface (Supplementary Fig. S8A–H).

Cpd-32 is composed of a bromopyridine-carboxamide, a pentafluropropyl and a chloroaniline moiety forming an acylaminal scaffold (Figs. 1A, 2C, and 2D). The nitrogen atoms of the acylaminal form a hydrogen bond with the side chain of S117^3.31^, which helps the recognition of Cpd-32 to S1P_3_ (Fig. 2C and 2D). Mutating this residue to alanine substantially reduced the inhibitory potency of Cpd-32 by ˜33-fold, which almost eliminates the inhibition by this antagonist (Fig. 2E and Supplementary Table S2).

The bromopyridine of Cpd-32 moiety binds a hydrophobic pocket (helices II-IV) involving L87^2.52^, A91^2.56^, V120^3.34^, W162^4.50^, and F166^4.54^ (Fig. 2C-D), and mutating these residues appeared 8-30-fold of ligand binding (Fig. 2E, 2F and Supplementary Table S2). The pentafluropropyl moiety of Cpd-32 fits into a sub-pocket shaped by helixes Ⅱ and Ⅲ (A91^2.56^, V94^2.59^, and L113^3.27^) (Fig. 2C and 2D). SAR data suggested that replacement of the pentafluoropropyl group of Cpd-32 with a longer dodecafluoropropyl group or a rigid chlorobenzene substituent results in severe loss of antagonistic potency by 45- or 88-fold (Nguyen et al., 2012), probably due to steric clashes with the side chains of A91^2.56^ or V94^2.59^ (Fig. 2E and Supplementary Table S2).

Cpd-32’s chloroaniline group engages helices III and IV via hydrophobic interactions with L113^3.27^, V120^3.34^, F166^4.54^, and A170^4.58^ (Fig. 2C and 2D). A halogen bond between its chlorobenzene and F166^4.54^’s backbone carbonyl further stabilizes binding. SAR data showed that replacement of the chloroaniline by dimethylcyclohexane results in a dramatic drop (516-fold) in ligand potency (Nguyen et al., 2012). In addition, TRUPATH assay suggests the replacement of the side chain of V120^3.34^ with a bulky side chain of tryptophan resulted in a complete loss of inhibitory potency of Cpd-32, which might be caused by the introduction of the steric hindrance with this ligand (Fig. 2F and Supplementary Table S2). Different from Cpd-32, CYM52581 features a bromopyridine, a dichlorobenzene, and a fluorobenzonitrile residue connected by a central carboxamide moiety (Fig. 1A, 2G, and 2H). Unlike Cpd-32, its carboxamide orients closer to helix III (Supplementary Fig. S8G and S8H), enabling its carbonyl oxygen atom of the carboxamide core of CYM52581, instead of a nitrogen atom in the acylaminal scaffold of Cpd-32, forms a hydrogen bond with S117^3.31^ (Fig. 2G and 2H).

The bromopyridine moiety of CYM52581 also binds to the hydrophobic pocket formed by helices II-IV (Fig. 2G). Replacement of S117^3.31^, V120^3.34^, and F166^4.54^ in the pocket to alanine reduced the affinity of CYM52581, mirroring Cpd-32 effects (Fig. 2I and Supplementary Table S2). However, its bromopyridine shifts closer to helix II, weakening F166^4.54^’s interaction (Fig. 2G). Indeed, mutating F166^4.54^ to alanine showed less impact on ligand potencies (9-fold vs. 4-fold), highlighting the importance of the hydrophobic interactions in ligand binding (Fig. 2I and Supplementary Table S2). Its *para*-chlorine rotates ˜60° and points to upper helix IV compared with Cpd-32 (Supplementary Fig. S8G and S8H), forming a halogen bond interaction with the backbone carbonyl of G169^4.57^ (Fig. 2G and 2H). SAR data showed a 7-fold loss of antagonistic effect when the dichlorobenzene is replaced with a benzyl substituent (Roberts et al., 2019). In addition, the *meta-*chlorine atom forms a hydrophobic interaction with F166^4.54^, and alanine mutation of this residue exhibited a 4-fold potency loss according to the TRUPATH assay (Fig. 2G–I and Supplementary Table S2). This aligns well with previous SAR studies which showed that substitution of dichlorobenzene with chlorobenzene decreased the inhibition by 1.6-fold (Roberts et al., 2019).

The bitopic antagonist SPM-242 and allosteric antagonists Cpd-32 or CYM52581 are preventing the receptor activation through different mechanisms. The bitopic antagonist SPM-242 is most likely to inhibit the receptor activation by occupying the endogenous ligand pocket and preventing the access of the agonist (Supplementary Fig. S6A and S6B). As described above, the zwitterionic head of SPM-242 binds to the orthosteric pocket formed by N terminus, helices Ⅱ-Ⅲ, and ECL1, which forms direct competition with the phosphonate and amine groups of the endogenous ligand S1P (Supplementary Fig. S6B). In addition, the hydrophobic tail of SPM-242 occupies the tunnel formed by helices Ⅰ, Ⅱ, and Ⅶ (Supplementary Fig. S6C and S6D). Unlike allosteric antagonists (Cpd-32/CYM52581), SPM-242 likely obstructs ligand access via membrane pathways, analogous to S1P ligand binding (Supplementary Fig. S6E).

On the contrary, the allosteric ligands Cpd-32 or CYM52581 bind helix III, restricting the inter-helical rearrangement critical for S1P3 activation. Structural superimposition of S1P_3_ in the active and inactive states showed moderate structural shifts (Cα r.m.s.d. 1.62 Å), including helix III shifts to the helix II in the active (Fig. 2K). This displaces S117^3.31^ (2.6 Å outward) and V120^3.34^, collapsing the allosteric ligand-binding cavity (Fig. 2L–O). Vice versa, ligand binding locks helix III in inactive state and antagonizes the receptor by preventing one of the key conformational changes during activation (Zhao et al., 2021). In addition, the side chain of M118^3.32^ flips away from helix II and points to the center of the helical bundle upon receptor inhibition, while the side chain of Y92^2.57^ also undergoes the notable rotation towards the center of the helical bundle from helix VII (Fig. 2P). As a result, the side chains of Y92^2.57^ and M118^3.32^ form hydrophobic interactions with transmission microswitch W256^6.48^, which mimic the antagonist and further prevent the receptor activation, stabilizing the receptor in the inactive state (Fig. 2P).

As the bitopic and allosteric ligands bind to two completely different pockets of S1P_3_ and inhibit the receptor in distinct mechanisms, the simultaneous binding of these ligands is synergistic. The binding of allosteric ligands, which stabilize the receptor in the inactive state, further facilitates the binding of bitopic ligand SPM-242. Thermo-stability assays show SPM-242 alone minimally effects stability, while Cpd-32/ CYM52581 increase stability by ˜8°C. Co-binding both ligands further boosts stability by ˜12°C (Supplementary Fig. S2A and S2B).

S1P_3_ and S1P_1_ share high sequence homology (49% identity; Cα r.m.s.d. 0.89 Å), with only 3 orthosteric pocket residue differences (Michael A. Hanson et al., 2012). However, the bitopic binding site of SPM-242 in S1P_3_ provides the second binding site, which is relatively divergent, and there are 4 residue differences within the hydrophobic pocket composed of a total of 6 amino acids (Supplementary Figs. S6A and S9). The high sequence variety might serve for the high selective ligand design, and indeed SPM-242 exhibits a high selectivity of S1P_3_ over S1P_1_ (Jo et al., 2012). The four residues, I96^2.61^, Q281^7.36^, W282^7.37^, and I284^7.39^, might be the determinants of the selectivity for SPM-242. Site-directed mutagenesis showed that the replacement of residues such as I284^7.39^ with corresponding residue leucine in S1P_1_ decreased the antagonistic effect by 11-fold in the presence of SPM-242 (Supplementary Table S2).

Besides the bitopic site, the allosteric site of S1P3, though conserved across S1P receptors, exhibits greater sequence divergence (5/13 residue differences) enabling selective ligand design. CYM52581 achieves 140-fold selectivity toward S1P_3_ over S1P_1_. Among the 5 residues, V94^2.59^, M98^2.63^, and V110^3.34^ formed a hydrophobic environment to accommodate the fluorobenzonitrile moiety of CYM52581, and mutating them to the counterpart residues greatly impaired the binding of allosteric ligands (Fig. 2G, 2H, 2J and Supplementary Table S2). For instance, mutating V110^3.34^ to glutamine or leucine of other S1P receptors dramatically decreases the antagonistic effect of CYM52581 by 75- or 56-fold, potentially due to sterically hindering the CYM52581 binding of larger side chains (Supplementary Table S2). In addition, V94^2.59^A or M98^2.63^L mutants, which possibly weaken the hydrophobic interactions, also decreased the antagonistic effect of CYM52581 by 2- or 7-fold (Fig. 2J and Supplementary Table S2). The other two different residues in the allosteric site, F166^4.45^ and A170^4.58^, form hydrophobic interactions with the chlorine atoms of chlorobenzene and serve for the ligand selectivity of S1P_3_ (Fig. 2G and 2H). Mutating these residues to corresponding residues as in other S1P receptors showed ˜2-fold decrease of the CYM52581 binding (Fig. 2I and Supplementary Table S2). Thus, the high sequence variation within the allosteric pocket might serve for the specific ligand design targeting each of the S1P receptors.

In this study, we have determined two distinct crystal structures of inactive S1P3 receptor in complex with the bitopic antagonist SPM-242 as well as the allosteric antagonist Cpd-32 or CYM52581. Additionally, we also solved the cryo-EM structures of S1P2 and S1P3 in complex with G_i_ proteins. These structures offer a comprehensive understanding of the unique binding mechanism of the bitopic ligand SPM-242 and the process of receptor activation. Unlike previous S1P receptor structures, SPM-242 simultaneously occupies the orthosteric site and blocks the ligand access entrance.

## Supplementary data

Supplementary data is available at *Protein & Cell Journal* online https://doi.org/10.1093/procel/pwaf068.

## Supplementary Material

pwaf068_Supplementary_Data

## Data Availability

Atomic coordinates for the structures of S1P3–SPM-242–Cpd-32, and S1P3–SPM-242–CYM52581 have been deposited in the RCSB Protein Data Bank (PDB) under accession codes 9W0H and 9W0L. Atomic coordinates and cryo-EM density maps for the structures of inactive S1P2-Gi and S1P3-Gi have been deposited in the RCSB Protein Data Bank (PDB) under accession codes 9W0M and 9W0O, and the Electron Microscopy Data Bank (EMDB) under accession codes EMD-65508 and EMD-65510.
