# Supplementary material for: Structural basis of allosteric and bitopic ligands binding in sphingosine-1-phosphate receptors 2 and 3
Source: Protein Cell. 2025 Aug 20;17(7):662–8. doi: 10.1093/procel/pwaf068 (PMC13340923; doi:10.1093/procel/pwaf068)
Supplement: pwaf068_Supplementary_Data [file pwaf068_supplementary_data.docx]

**Supplementary Materials**

**Structural basis of allosteric and bitopic ligands binding in sphingosine-1-phosphate receptors 2 and 3**

Yanhong Wu^1,2,7^, Qiuru Chen^1,7^, Hongyu Wang^1,2^, Kezhen Liu^1,2^, Jiaxin Wei^1,2^, Mu Wang^1,7^, Kun Chen^1,2^, Ya Zhu^4^, Shuo Han^1^, Cuiying Yi^1^, Limin Ma^1^, Gisela Schnapp^5^, Alexander Pautsch^5^, Christian Gnamm^5^, Matthias Grauert^5^, Esther Schmidt^6^, Qiuxiang Tan^1^*, Beili Wu^1,2,3,7^*, Qiang Zhao^1,2,8^*

^1^State Key Laboratory of Drug Research and State Key Laboratory of Chemistry Biology, Shanghai Institute of Materia Medica, Chinese Academy of Sciences, 555 Zuchongzhi Road, Pudong, Shanghai, China

^2^University of Chinese Academy of Sciences, 19A, Yuquan Road, Beijing, China

^3^School of Pharmaceutical Science and Technology, Hangzhou Institute for Advanced Study, UCAS, Hangzhou, Zhejiang, China.

^4^Lingang Laboratory, Shanghai 200031, China.

^5^Boehringer-Ingelheim Pharma GmbH & Co. KG, Department of Medicinal Chemistry, Birkendorfer Str. 65, 88397 Biberach, Germany.

^6^Boehringer-Ingelheim Pharma GmbH & Co. KG, Department of Drug Discovery Sciences, Birkendorfer Str. 65, 88397 Biberach, Germany.

^7^School of Life Science and Technology, ShanghaiTech University, 393 Hua Xia Zhong Road, Pudong, Shanghai, China

^8^Zhongshan Institute of Drug Discovery, SIMM, CAS, Zhongshan, Guangdong, China.

^9^These authors contributed equally: Yanhong Wu, Qiuru Chen.

*Correspondence: qxtan@simm.ac.cn; beiliwu@simm.ac.cn; zhaoq@simm.ac.cn.

**MATERIALS AND METHODS**

**Synthesis of SPM-242, Cpd-32 and CYM52581**

The compounds used in this study (SPM-242, Cpd-32 and CYM52581) were synthesized by WuXi AppTec as described in the literature (for details see the Supporting Information).

**Construct design for S1P_3_ crystal structures**

To improve the protein homogeneity, 35 residues at the C terminus (N343-N378) and 10 residues (M1-Q10) at the N terminus of the receptor were truncated, respectively. Besides, xylanase (PDB: 2B45) fusion partner(Chun et al. 2012) replaced the receptor residues S224-N231 of ICL3. We also introduced two thermostabilizing mutations H137^3.51^Y and C84^2.49^S in order to improve the protein yield and stability using overlapping PCR. The engineered construct was cloned into a modified pFastBac1 vector (Invitrogen) containing hemagglutinin (HA) signal sequence followed by a Flag-tag at the N terminus and a PreScission Protease recognition site and a 10 × His-tag at the C terminus.

**Expression and purification of S1P_3_ in complex with antagonists**

High-titer recombinant baculovirus was generated using Bac-to-Bac Baculovirus Expression System (Invitrogen). *Spodoptera frugiperda* (*sf*9) insect cells at a density of 2×10^6^ cells per mL were infected at a multiplicity of infection (MOI) of 5. Cells were harvested by centrifugation 48 h post-transfection, and stored at −80 ℃ until use.

The insect cells expressing S1P_3_ were treated with hypotonic buffer containing 10 mM HEPES, pH 7.5, 20 mM KCl, 10 mM MgCl_2_, and EDTA-free protease inhibitor cocktail (Roche) by dounce homogenization. Then, the cell pellets were disrupted by repeated washing and centrifugation in a high osmotic buffer containing 10 mM HEPES, pH 7.5, 20 mM KCl, 10 mM MgCl_2_, and 1 M NaCl. The membrane was washed with hypotonic buffer to remove the high concentration of NaCl. The membrane was resuspended in hypotonic buffer with 30% glycerol and flash frozen in liquid nitrogen, and then stored at −80 ℃.

The membrane was thawed on ice and incubated with 2 mg mL^-1^ iodoacetamide (Sigma), EDTA-free protease inhibitor cocktail (Roche), 10 μM SPM-242, 10 μM Cpd-32, or 10 μM CYM52581 at 4 ℃ for 1h, and was solubilized in a buffer containing 50 mM HEPES, pH 7.5, 300 mM NaCl, 1% (w/v) *n*-dodecyl-β-D-maltopyranoside (DDM, Anatrace) and 0.2% (w/v) cholesterol hemisuccinate (CHS, Sigma) at 4 ℃ for 2-3 h. The solubilized membrane was centrifuged at 160,000 *g* for 30 min to remove the membrane debris and then the TALON IMAC resin (Clontech) was added to the supernatant supplemented with 5 mM imidazole and incubated overnight at 4 ℃. The resin was washed with 20 column volumes of wash buffer Ⅰ containing 30 mM imidazole, 10 μM SPM-242, 10 μM Cpd-32 or 10 μM CYM52581, 25 mM HEPES, pH 7.5, 150 mM NaCl, 0.05% (w/v) DDM, 0.01% (w/v) CHS, 10% (v/v) glycerol. Then, the complex was washed in 10 column volumes of ATP wash buffer Ⅱ containing 5 mM ATP sodium salt (Sigma), 10 mM MgCl_2_, 10 μM SPM-242, 10 μM Cpd-32 or 10 μM CYM52581, 25 mM HEPES, pH 7.5, 150 mM NaCl, 0.05% (w/v) DDM, 0.01% (w/v) CHS, 10% (v/v) glycerol, followed by 10 column volumes of wash buffer Ⅲ containing 10 μM SPM-242, 10 μM Cpd-32 or 10 μM CYM52581, 25 mM HEPES, pH 7.5, 150 mM NaCl, 0.05% DDM, 0.01% CHS, 10% (v/v) glycerol. Protein was eluted by 5 column volumes of buffer composing of 25 mM HEPES, pH 7.5, 150 mM NaCl, 0.025% (w/v) DDM, 0.01% (w/v) CHS, 10 % (v/v) glycerol, 300 mM imidazole, 10 mM Cpd-32 or 10 μM CYM52581, 20 μM SPM-242. A PD MiniTrap G-25 column (GE Healthcare) was used to remove imidazole, and then the complex was treated with His-tagged PreScission Protease (home-made) and His-tagged PNGase (home-made) overnight to remove the C-terminal His-tag and glycosylation. The PreScission Protease and PNGase were removed by incubating with the TALON IMAC resin for 4 h at 4 ℃. The final complex was concentrated to minimal volume with a 100 kDa molecular weight cut-off Vivaspin centrifuge concentrator (Sartorius). The protein quality was tested by analytical size-exclusion chromatography (aSEC) and SDS-page.

**Crystallization**

The concentrated ternary complexes were reconstituted into LCP through mixing monoolein lipid (Anatrance) and cholesterol (Sigma) in a ratio of 10:1 (w/w) with protein in 2:3 (w/w) using the syringe mixer(Caffrey and Cherezov 2009). The protein-lipid mixture is dispensed onto the 96-well sandwich plate in 30- to 40-nl and overlaid with the 800 nl crystallization solution using a Gryphon LCP robot (Art Robbins Instruments). Crystals appeared after two days and grew into the biggest size in two weeks at room temperature. The optimized crystals of S1P_3_ in complex with SPM-242 and Cpd-32 appeared in crystallization solution containing 100 mM Tris-HCl, pH 8.0, 18% PEG500DME, 50 mM NaCl, 50 mM Li_2_SO_4_, 12 mM ATP. Crystals of S1P_3_ with SPM-242 and CYM52581 grew in 100 mM Tris-HCl, pH 8.0, 20% PEG600, 60 mM Li_2_SO_4_, 15% DMSO. The crystals were harvested by micro-loops (MiTeGen) and flash frozen in liquid nitrogen.

**Data procession and refinement for S1P_3_ crystal structures**

X-ray diffraction data of S1P_3_ with SPM-242 and Cpd-32 were collected at the Spring-8 beam line 41XU, Hyogo, Japan, using an EIGER 16M detector and beamline 45XU, using a Pilatus 6M detector (X-ray wavelength 1.0000 Å). Diffraction data of the S1P_3_ complex with SPM-242 and Cpd-32 were integrated and scaled with HKL2000 (Otwinowski and Minor 1997). Initial phase information was obtained through the molecular replacement (MR) in phaser(McCoy et al. 2007) with S1P_1_ (PDB code: 3V2Y) and xylanase (PDB code: 2B45) as search models. Refinement was performed by REFMAC5(Vagin et al. 2004), autoBUSTER (Smart et al. 2012), and PHENIX (Afonine et al. 2012) and subsequent rebuilding was carried out in COOT (Emsley et al. 2010) with both 2|*F*o|-|*F*c| and |*F*o|-|*F*c| maps.

X-ray diffraction data of S1P_3_ bound to SPM-242 and CYM52581 was collected at the SPring-8 beamline 45XU, using a Pilatus 6M detector (X-ray wavelength 1.0000Å). The ZOO automatic data collection and processing system was used. The diffraction data was solved by XDS(Kabsch 2010). In brief, the whole S1P_3_ in complex SPM-242 and Cpd-32 structure was used as a search model for MR. Both structures were carefully refined. The Ramachandran plot analysis of the final structure with Molprobity indicates that 100% residues are in either favored (S1P_3_–SPM-242–Cpd-32, 95.36%; S1P_3_–SPM-242–CYM52581, 95.15%) or allowed regions (S1P_3_–SPM-242–Cpd-32, 4.64%; S1P_3_–SPM-242–CYM52581, 4.85%) (no outlier).

**Construct design and expression of S1P_2_/S1P_3_**–**Gα_i_ complex**

The human S1P_2_ receptor is truncated by 40 residues at C-terminus to improve the protein yield and homogeneity. To facilitate expression and purification, the coding sequence of human S1P_2_ (residues 1-353) and full-length S1P_3_ (residues 1-378) were cloned into a modified pFastBac1 vector (Invitrogen) with a hemagglutinin (HA) signal sequence, an N-terminal FLAG tag, and a C-terminal PreScission Protease site followed by a 2 × Strep-tag (WSHPQFEKGGGSGGGSGGSAWSHPQFEK). The dominant-negative human Gα_i1_ gene was generated by introducing five mutations (S47C, G202T, G203A, E245A, and A326S) in order to decrease the affinity of nucleotide binding and increase the stability of Gα :Gβγ complex(Liang et al. 2018). Both human Gβ_1_ with an N-terminal 6 × His-tag and Gγ_2_ subunits were integrated into the pFastBac Dual vector (Invitrogen). Human S1P_3_, S1P_2,_ and G_i1_ heterotrimer were co-expressed in High-Five insect cells using the Bac-to-Bac Baculovirus Expression System (Invitrogen). The High-Five cells were infected at a cell density of 1.5 × 10^6^ cells per mL with three separate virus preparations for modified S1P_2_, Gα_i1,_ and Gβ_1_γ_2_ with high-titer at a ratio of 1:2:2. While for the S1P_3_–G_i_ complex, the cells were infected virus at a MOI ratio of 1:1:1 for the S1P_3_, Gα_i1_ and Gβ_1_γ_2_. Cells were cultured at 27 °C and harvested 48 h after infection by centrifugation and stored at −80°C until use.

**Purification of S1P_2_/S1P_3_**–**G_i_ complex**

The 250 ml S1P_3_–G_i_ or S1P_2_–G_i_ co-expression cell pellets were thawed and lysed in the hypotonic buffer of 25 mM HEPES, pH 7.5, 150 mM NaCl, 10 mM MgCl_2_, 100 μg mL^-1^ protease inhibitor and homogenized by douncing. For the S1P_3_–G_i_ complex, 25 µM CYM-5541 was added. In the case of S1P_2_–G_i_ complex, 25 µM CYM-5520 was added. Both complexes were incubated at room temperature for 1 h in the presence of apyrase (New England Bio-Labs) at a final concentration of 25 mU mL^-1^. Then, 0.5%(w/v) DDM and 0.1% (w/v) CHS were added to solubilize the membrane at 4 °C for 3 h. The supernatant was collected by centrifugation at 30,000 *g* for 30 min and incubated with pre-equilibrated Strep-Tactin resin (IBA Lifesciences) at 4 °C overnight.

For the S1P_3_–G_i_ complex, the Strep-Tactin resin (IBA Lifesciences) was washed with 10 column volumes of washing buffer containing 25 mM HEPES, pH 7.5, 150 mM NaCl, 10 mM MgCl_2_, 0.05% (w/v) DDM, 0.01% (w/v) CHS and 25 µM CYM-5541. Then the detergent was exchanged on column with 10 column volumes of exchange buffer containing 25 mM HEPES, pH 7.5, 150 mM NaCl, 10 mM MgCl_2_, 0.8% (w/v) lauryl maltose neopentyl glycol (LMNG, Anatrace), 0.01% (w/v) CHS, 0.27% (w/v) glyco-diosgenin (GDN, Anatrace) and 25 µM CYM-5541 at 4 °C for 2 h. The resin was further washed with 20 column volumes of buffer containing 25 mM HEPES, pH 7.5, 150 mM NaCl, 10 mM MgCl_2_, 0.01% (w/v) LMNG, 0.0033% (w/v) GDN and 25 µM CYM-5541. The complex was eluted with 5 column volumes of elution buffer containing 200 mM Tris-HCl, pH 8.0, 150 mM NaCl, 10 mM MgCl_2_, 0.01% (w/v) LMNG, 0.0033% (w/v) GDN, 50 mM biotin (Sigma) and 25 µM CYM-5541, followed by concentration to 0.5 mL using an Amicon Ultra Centrifugal Filter (MWCO100kDa). The complex was subjected to a Superdex 200 Increase 10/300 column (GE Healthcare) to separate complex from contaminants. Pre-equilibrated with AKTA buffer containing 25 mM HEPES, pH 7.5, 150 mM NaCl, 10 mM MgCl_2_, 0.0008% (w/v) LMNG, 0.00027% (w/v) GDN and 0.00014% CHS, 5 μM CYM-5541. The complex peak fractions were collected and concentrated to 1.55 mg mL^-1^ for electron microscopy experiments.

The S1P_2_–G_i_ complex purification was performed using the same procedure as described before, there are some details different from the S1P_3_–G_i_ complex. The Strep-Tactin resin (IBA Lifesciences) with immobilized S1P_2_–G_i_ complex was washed with 10 column volumes of washing buffer mentioned above and 25 µM CYM-5520. Then the detergent was exchanged on column with 10 column volumes of exchange buffer containing 25 mM HEPES, pH 7.5, 150 mM NaCl, 10 mM MgCl_2_, 0.25% (w/v) GDN and 25 µM CYM-5520 at 4 °C for 2 h. The resin was further washed with 20 column volumes of buffer containing 25 mM HEPES, pH 7.5, 150 mM NaCl, 10 mM MgCl_2_, 0.01% (w/v) GDN and 25 µM CYM-5520. The complex was eluted with 5 column volumes of elution buffer containing 200 mM Tris-HCl, pH 8.0, 150 mM NaCl, 10 mM MgCl_2_, 0.01% (w/v) GDN, 50 mM biotin and 25 µM CYM-5520. The purified complex was concentrated, then injected onto a Superdex200 10/300 GL column (GE Healthcare). Pre-equilibrated with AKTA buffer containing 25 mM HEPES, pH 7.5, 150 mM NaCl, 10 mM MgCl_2_, 0.01% (w/v) GDN and 5 μM CYM-5520. Peak fractions were concentrated to 7.8 mg/mL using 100kDa cutoff concentrator for electron microscopy studies. These final samples were evaluated by SDS–PAGE and analytical size-exclusion chromatography.

**Cryo-EM grid preparation**

The purified CYM-5520–S1P_2_–G_i_ or CYM5541–S1P_3_–G_i_ complex were applied to glow-discharged holey carbon grids (Quantifoil R1.2/1.3, 200 mesh). The grids were blotted for 3 s under 100% humidity at 4 °C and then vitrified by plunging into liquid ethane using a Vitrobot Mark IV (Thermo Fisher Scientific). Cryo-EM images were collected on a Titan Krios equipped with a Gatan K3 Summit direct electron detector. The microscope was operated at 300 kV accelerating voltage, at a nominal magnification of ×29,000 in counting mode, corresponding to a pixel size of 1.045 Å. In total. The total exposure time was set to 3 s with intermediate frames recorded every 0.075 s, resulting in an accumulated dose of 70 electrons per Å^2^. Automated single-particle data acquisition was performed with SerialEM(Mastronarde 2005).

**Image processing and 3D reconstruction**

For the S1P_3_–G_i_ complex, a total of 5,405 images were collected, followed by beam-induced motion correction and contrast transfer function (CTF) determination. All 2,237,371 particles were subjected to two rounds of reference-free 2D classification to discard false-positive particles. An ab initio model generated by RELION3.0 was used as an initial reference model for 3D classification. A subset of 1,119,753 particles was selected for another round of 3D classification that focused the alignment on the complex. The best-looking dataset of 669,267 particles was subjected to 3D auto-refinement, resulting in an initial 3.2 Å density map. A final 3.0 Å map was sharpened by post-process with a B-factor of -103.75 Å^2^. The local resolution map was calculated using RELION3.0. Surface coloring of the density map was performed using PyMOL (http://pymol.org/2/).

For the S1P_2_–G_i_ complex, a total of 11,178 images were collected. The dataset was subjected to motion correction, CTF estimation, auto-picking, 2D classification, and 3D classification in RELION 3.0 (Scheres 2012). About 13,512,072 particles were extracted for further 3D processing. After auto-refinement and further Bayesian polishing applied to these particles in RELION3.0 improved the density map with a nominal resolution of 3.8 Å.

**Model building and refinement**

G_i_ protein heterotrimer in FPR2–G_i_ (PDB code: 6OMM) complex was used to initial template. For the S1P_3_–G_i_, the S1P_3_ receptor in the S1P_3_–SPM-242–Cpd-32 crystal structure complex was used as the starting model. In the case of the S1P_2_–G_i_ complex, the initial homology model of active S1P_2_ was generated by the Swiss-model using S1P_1_ (PDB code 3V2Y) (Waterhouse et al. 2018). All models were docked into the EM density map using Chimera, followed by iterative manual adjustment and rebuilding in COOT and real space refinement using phenix. real_space_refine in Phenix. The model statistics was validated using MolProbity. Structural figures were prepared in PyMOL (http://pymol.org/2/). The extent to which any model was overfitted during refinement was measured by refining the final model against one of the half-maps and by comparing the resulting map versus model FSC curves with the two half-maps and full model. The final refinement statistics are provided in Supplementary Table 2.

**Thermo-stability assay**

The thermo-stability of S1P_3_ in the presence of different ligands was determined by measuring the reaction with the thiol-specific fluorochrome 7-diethylamino-3-(4'-maleimidylphenyl)-4-methylcoumarin (CPM, Thermo). The test protein was purified by previous mentioned method without tag cleavage and PNGase treatment. The CPM dye stock was dissolved in DMSO at 4 mg mL^-1^ and kept at -80 ℃. The CPM dye stock is diluted 20-fold in a buffer containing 25 mM HEPES, pH 7.5,150 mM NaCl, 0.05% (w/v) DDM, 0.01% (w/v) CHS, 10% glycerol before use. The 3-5 μg test receptor bound to different ligands was mixed with 1 μl CPM dye in the same buffer to a final volume of 120 μl. The test proteins were incubated at room temperature for 20 min. Then the mixture was transferred to a quartz fluorometer cuvette (Starna Scientific Ltd) and heated from 20 ℃ to 75 ℃ at a rate of 1 ℃/min by the Cary Eclipse spectrofluorometer (Agilent Technologies). The CPM intensity (excitation 387 nm, emission 463 nm) was recorded with temperature change. The Tm value was calculated by fitting the data with Boltzmann sigmoidal equation in GraphPad Prism (GraphPad Software, San Diego, CA, USA).

**TRUPATH assay**

TRUPATH is based on biosensors to measure heterotrimeric G-protein dissociation assay(Olsen et al. 2020). The Flag-tagged wild-type and mutants were cloned into the pTT5 vector (Invitrogen). HEK293T cells were transfected by the plasmid in a 1:1:1:1 ratio of receptor: Gα_q_-RLuc8: Gβ_3_: Gγ_9_-GFP2 or receptor: Gα_i_-RLuc8: Gβ_3_: Gγ_9_-GFP2 (500 ng per construct) in 6-well dishes. HEK293T cells were cultured in Dulbecco’s Modified Eagle Medium (DMEM, Wisent) supplemented with 8% FBS (Wisent) and 1% penicillin-streptomycin (Wisent) and maintained at 37 ℃ with 5% CO_2_. The surface expression level of receptor was measured by incubating with anti-FLAG FITC-labeled antibody (Sigma) using a Guava flow cytometer (Millipore).

The following day, cells were harvested by 0.25% Trypsin-EDTA (Thermo Fisher Scientific) and plated in a poly-d-lysine-coated 96-well assay plates (Beyotime) at a cell density of 2,5000 cells per well and grew for an additional 24h at 37 ℃ with 5% carbon dioxide. For the TRUPATH assay, the medium was replaced with 60 μl buffer containing 20 mM HEPES, pH 7.4, 0.1% BSA (Sigma), and 1 ⅹ Hank’s balanced salt solution (HBSS) buffer. Coelenterazine 400a (Nanolight Technologies) was freshly prepared and added to a final concentration of 50 μM. After 5 min of incubation at room temperature, the baselines were read by Synergy H1 microplate reader (BioTek Technologies) with 395 nm (Rluc8-coelenterazine 400a) and 510 nm (GFP2) emission filters. After the equilibration period, cells were treated with 30 μl corresponding increasing ligand concentration. For the antagonist assay, 20 μM S1P was used to dilute the different concentrations of antagonists SPM-242, Cpd-32 and CYM52581, and simultaneously added to the cells. The BRET ratios were computed as the ratio of the GFP2 emission to the Rlu8 emission. The data were analyzed with dose-response stimulation in GraphPad Prism 9 (GraphPad Software, San Diego, CA, USA). Non-liner curve fit was performed using a three-parameter logistic equation [log (agonist vs response)] or [log (antagonist vs response)].

**cAMP assay**

Potencies of S1P_3_ inhibitors were determined in CHO-K1 cells stably expressing the human S1P_3_ receptor via detection of intracellular cAMP content. Specific inhibition of S1P-stimulated cAMP decrease was measured using Cisbio HTRF technology, according to the manufacturer.

**REFERENCES**

Afonine, P. V., R. W. Grosse-Kunstleve, N. Echols, J. J. Headd, N. W. Moriarty, M. Mustyakimov, T. C. Terwilliger, A. Urzhumtsev, P. H. Zwart, and P. D. Adams. 2012. 'Towards automated crystallographic structure refinement with phenix.refine', *Acta Crystallogr D Biol Crystallogr*, 68: 352-67.

Caffrey, M., and V. Cherezov. 2009. 'Crystallizing membrane proteins using lipidic mesophases', *Nat Protoc*, 4: 706-31.

Chun, E., A. A. Thompson, W. Liu, C. B. Roth, M. T. Griffith, V. Katritch, J. Kunken, F. Xu, V. Cherezov, M. A. Hanson, and R. C. Stevens. 2012. 'Fusion partner toolchest for the stabilization and crystallization of G protein-coupled receptors', *Structure*, 20: 967-76.

Emsley, P., B. Lohkamp, W. G. Scott, and K. Cowtan. 2010. 'Features and development of Coot', *Acta Crystallogr D Biol Crystallogr*, 66: 486-501.

Kabsch, Wolfgang. 2010. 'XDS', *Acta Crystallographica Section D*, 66: 125-32.

Liang, Yi-Lynn, Peishen Zhao, Christopher Draper-Joyce, Jo-Anne Baltos, Alisa Glukhova, Tin T. Truong, Lauren T. May, Arthur Christopoulos, Denise Wootten, Patrick M. Sexton, and Sebastian G. B. Furness. 2018. 'Dominant Negative G Proteins Enhance Formation and Purification of Agonist-GPCR-G Protein Complexes for Structure Determination', *ACS Pharmacology & Translational Science*, 1: 12-20.

Mastronarde, D. N. 2005. 'Automated electron microscope tomography using robust prediction of specimen movements', *J Struct Biol*, 152: 36-51.

McCoy, A. J., R. W. Grosse-Kunstleve, P. D. Adams, M. D. Winn, L. C. Storoni, and R. J. Read. 2007. 'Phaser crystallographic software', *J Appl Crystallogr*, 40: 658-74.

Olsen, R. H. J., J. F. DiBerto, J. G. English, A. M. Glaudin, B. E. Krumm, S. T. Slocum, T. Che, A. C. Gavin, J. D. McCorvy, B. L. Roth, and R. T. Strachan. 2020. 'TRUPATH, an open-source biosensor platform for interrogating the GPCR transducerome', *Nat Chem Biol*, 16: 841-49.

Otwinowski, Zbyszek, and Wladek Minor. 1997. '[20] Processing of X-ray diffraction data collected in oscillation mode.' in, *Methods in Enzymology* (Academic Press).

Scheres, S. H. 2012. 'RELION: implementation of a Bayesian approach to cryo-EM structure determination', *J Struct Biol*, 180: 519-30.

Smart, O. S., T. O. Womack, C. Flensburg, P. Keller, W. Paciorek, A. Sharff, C. Vonrhein, and G. Bricogne. 2012. 'Exploiting structure similarity in refinement: automated NCS and target-structure restraints in BUSTER', *Acta Crystallogr D Biol Crystallogr*, 68: 368-80.

Vagin, A. A., R. A. Steiner, A. A. Lebedev, L. Potterton, S. McNicholas, F. Long, and G. N. Murshudov. 2004. 'REFMAC5 dictionary: organization of prior chemical knowledge and guidelines for its use', *Acta Crystallogr D Biol Crystallogr*, 60: 2184-95.

Waterhouse, Andrew, Martino Bertoni, Stefan Bienert, Gabriel Studer, Gerardo Tauriello, Rafal Gumienny, Florian T. Heer, Tjaart A P de Beer, Christine Rempfer, Lorenza Bordoli, Rosalba Lepore, and Torsten Schwede. 2018. 'SWISS-MODEL: homology modelling of protein structures and complexes', *Nucleic Acids Research*, 46: W296-W303.

**
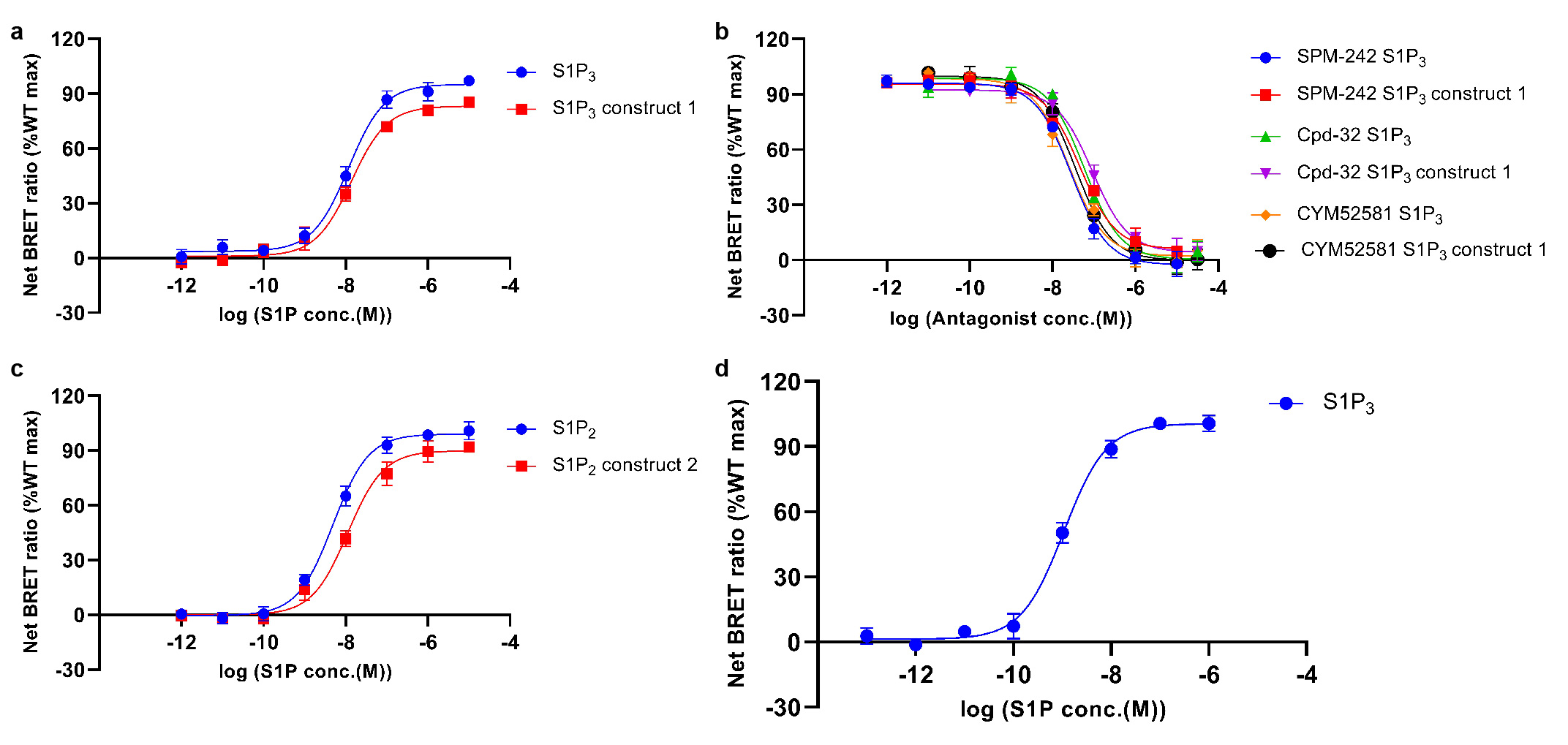
**

**Figure S1. TRUPATH assays of S1P_2_ and S1P_3_.** (A-D) Construct 1 indicates modified S1P_3_ used for crystallization trials, which removed ICL3-xylanase and retained all modification for crystallization to conduct the TRUPATH assay using G_q_ protein. WT, wild-type S1P_3_. Data are shown as mean ± SEM from three independent experiments performed in duplicate. (C and D), TRUPATH assays of wild-type (WT) S1P_2_, modified S1P_2_ and wild-type (WT) S1P_3_ using G_i_ protein. Construct 2 indicates C terminal truncation of S1P_2_ used for cryo-EM trials. Data are shown as mean ± SEM from three independent experiments performed in duplicate.

**
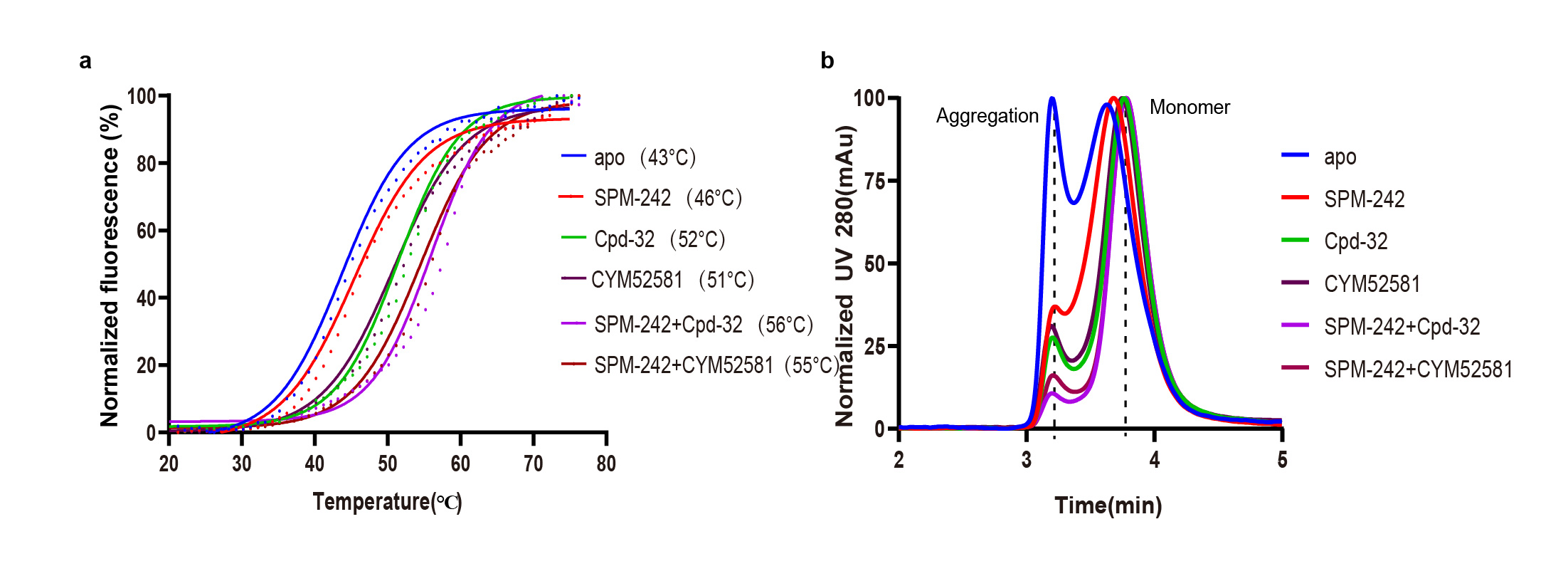
**

**Figure S2. Stability assays of S1P_3_ with SPM-242 and Cpd-32.** (A) CPM assays demonstrate higher stability of S1P_3_ with combined ligands in comparison of single ligand. The data were representative of three independent experiments. The allosteric ligand Cpd-32 further improved the thermal-stability. (B) The normalized results of analytical size-exclusion chromatography (aSEC) showed that adding two antagonists SPM-242 and Cpd-32 results in the lowest aggregation, which indicates the best protein homogeneity.

**
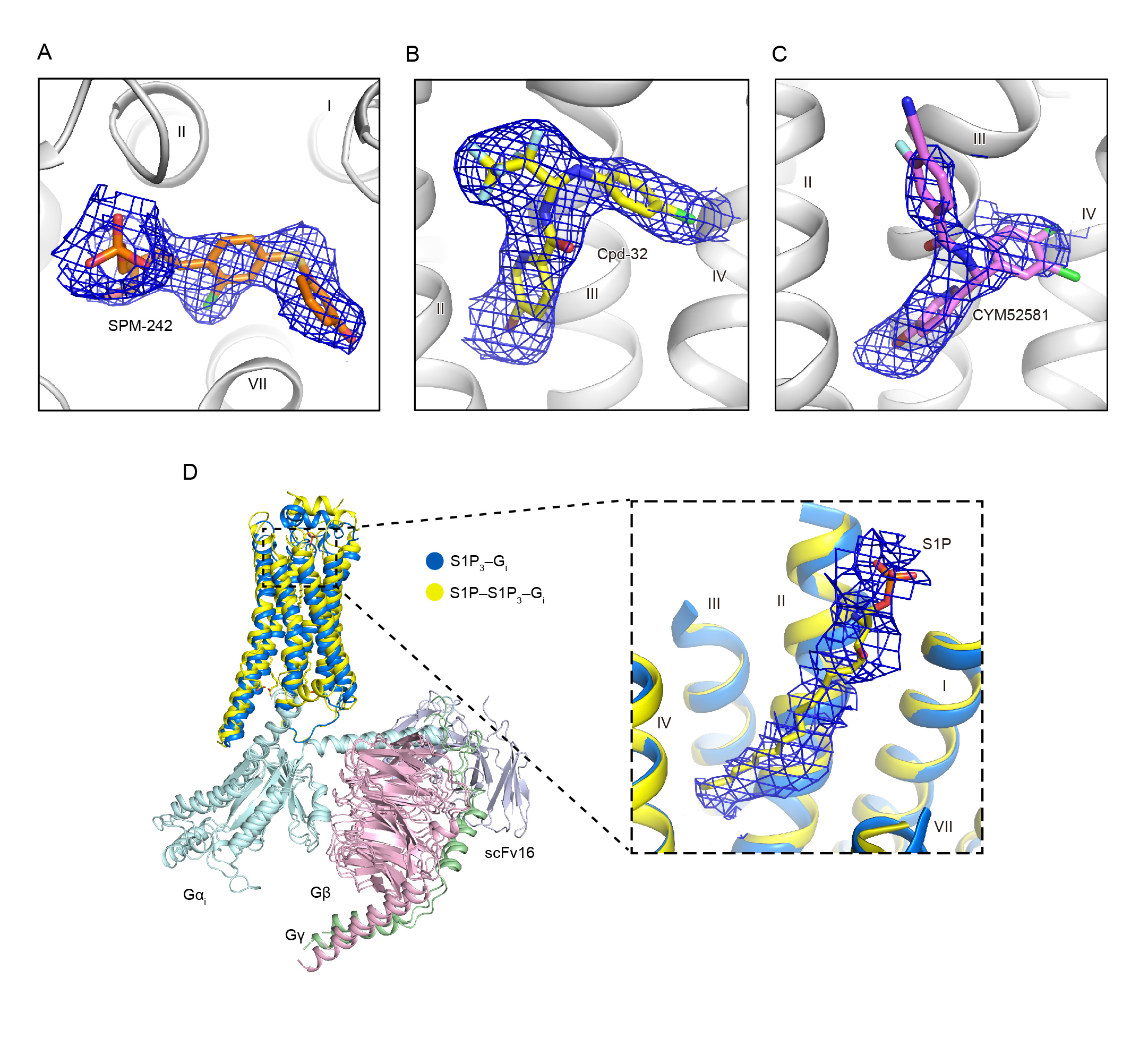
**

**Figure S3. Electron densities of SPM-242, Cpd-32 and CYM52581.** (A-C) 2*|F_o_|*-*|F_c_|* omit map of SPM-242, Cpd-32 and CYM52581. The S1P_3_ structure is shown in grey cartoon representation. SPM-242, Cpd-32 and CYM52581 are displayed as orange, yellow and violet sticks, respectively. Electron densities are contoured at 2.5 σ from 2*|F*_o_*|*-*|F*_c_*|* omit map and colored blue. (D) Superimposition of active S1P_3_ and S1P–S1P_3_–G_i_ complex. The structures of S1P_3_–G_i_ and S1P–S1P_3_–G_i_ (PDB ID:7EW3) are shown in cartoon representation, with receptors colored marine and yellow, respectively. S1P in S1P–S1P_3_–G_i_ is shown as yellow sticks and electron densities of S1P_3_–G_i_ are colored blue mesh. Gα_i_, Gβ, Gγ and scFv16 are colored light-cyan, light-pink, light-green, and light-blue, receptively.


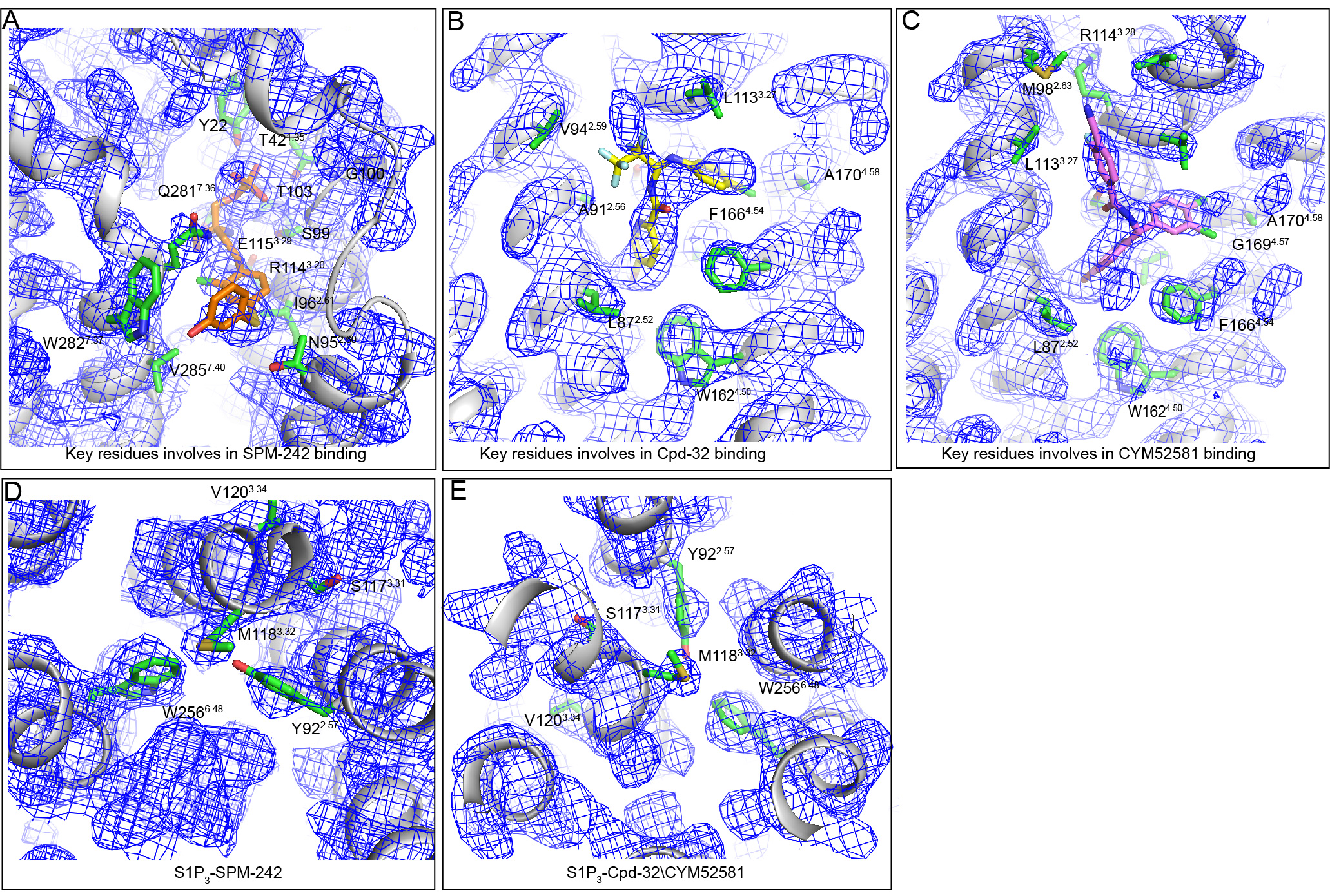


**Figure S4. Electron densities of receptor around SPM-242, Cpd-32 and CYM52581.** (A-C) Key residues mediating binding of SPM-242 (A), Cpd-32 (B), and CYM52581 (C) to the S1P3 receptor. The receptor structure is depicted as a grey cartoon with electron density maps shown in as blue mesh. Election densities are contoured at from at 1.8σ from 2|Fo|-|Fc| omit map. (D and E) Structural rearrangements during receptor activation. (D) Conformational shifts in residues proximal to SPM-242; (E) Shared activation-related movements in residues interacting with Cpd-32 and CYM52581.


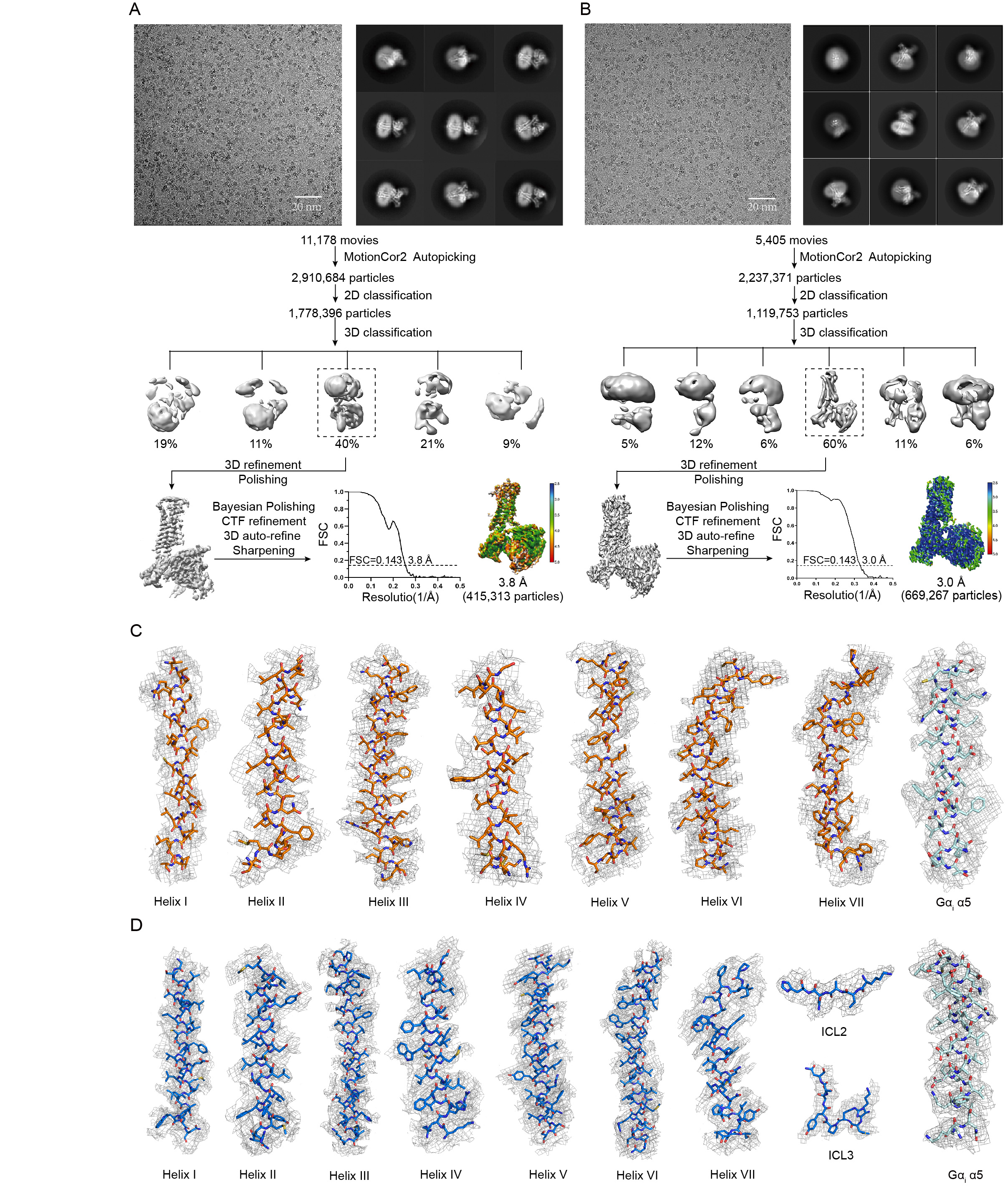


**Figure S5. Cryo-EM data procession and of the S1P_2_**–**G_i_ and S1P_3_**–**G_i_ complexes.** (A) Representative cryo-EM image and 2D averages of S1P_2_–G_i_. Workflow of cryo-EM data processing with cryo-EM map colored grey according to local resolution (Å) for S1P_2_–G_i_ complex. Gold-standard FSC curve of the S1P_2_–G_i_ complex, indicating an overall resolution at 3.8 Å. (B) Representative cryo-EM image and 2D averages of S1P_3_–G_i_. Workflow of cryo-EM data processing with cryo-EM map colored grey according to local resolution (Å) for S1P_3_–G_i_ complex. Gold-standard FSC curve of the S1P_3_–G_i_ complex, indicating an overall resolution at 3.0 Å. (C) Cryo-EM density map and the model of S1P_2_–G_i_ complex are shown for all transmembrane and α5 helix. S1P_2_ is shown in orange sticks representation. α5 helix of Gα_i_ is shown in light-cyan sticks representation. (D) Cryo-EM density map and the model of S1P_3_–G_i_ complex are shown for all transmembrane, ICL2 and ICL3 of S1P_3_ and α5 helix of G protein. S1P_3_ is shown in marine sticks representation. α5 helix of Gα_i_ is shown in light-cyan sticks representation.


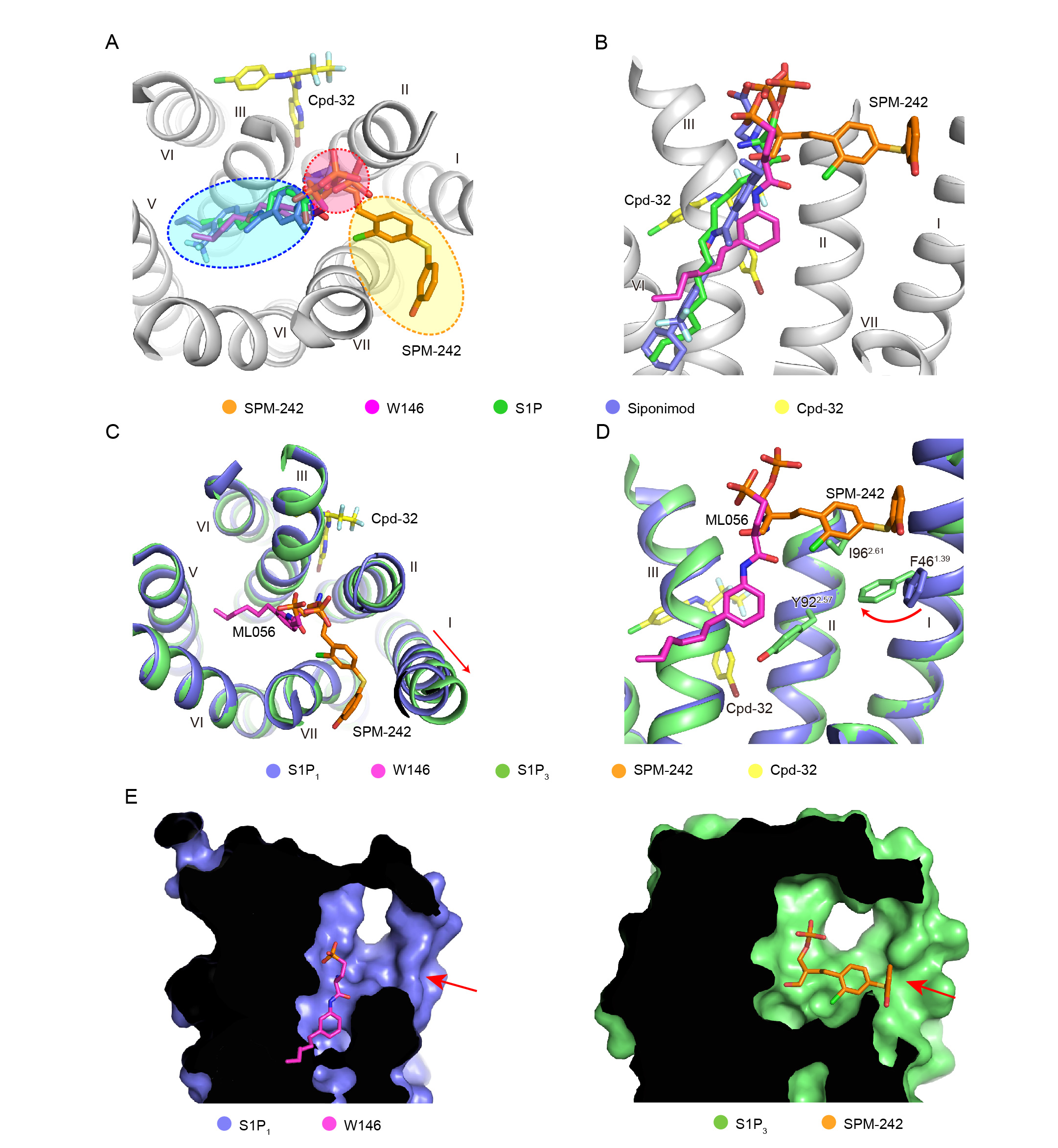


**Figure S6. Structural comparison of ligand-binding mode.** (A) Extracellular view. (B) Side view. Comparison of orthosteric ligand-binding mode in S1P_3_, S1P_1_, S1P_2_ and S1P_5_. The bitopic ligand SPM-242 of S1P_3_, antagonist W146 of S1P_1_ (PDB code: 3V2Y), endogenous ligand S1P of S1P_2_ (PDB code: 7T6B) and agonist Siponimod of S1P_5_ (PDB code: 7EW1) are shown in sticks representation and colored orange, magenta, green, and slate, respectively. The allosteric antagonist Cpd-32 was shown as yellow sticks. Only the helical bundle of S1P_3_ is shown in grey cartoon representation for clarity. The red ellipse indicates the binding-region shared by the polar head of those ligands, the orange ellipse indicates the second binding-pocket of SPM-242 in the S1P_3_ and the blue ellipse indicates the canonical binding-site for hydrophobic tail of W146, S1P and Siponimod. (C) Extracellular view. (D) Side view. The structures of inactive S1P_1_ and S1P_3_ are shown in the slate and lime cartoon representation, respectively. The ligands SPM-242, Cpd-32 and W146 are shown as orange, yellow, and magenta sticks, respectively. The conformational change of residues between S1P1 and S1P3 are shown as slate and lime sticks, respectively. The red arrows indicate the movement of helix I and the rotation of residues. (E) Cutaway view. The structures of S1P_3_ and S1P_1_ are shown in slate and lime surface representation, respectively. The ligands W146 and SPM-242 are shown as magenta and orange sticks, respectively. The red arrows indicate the putative ligands entrance.


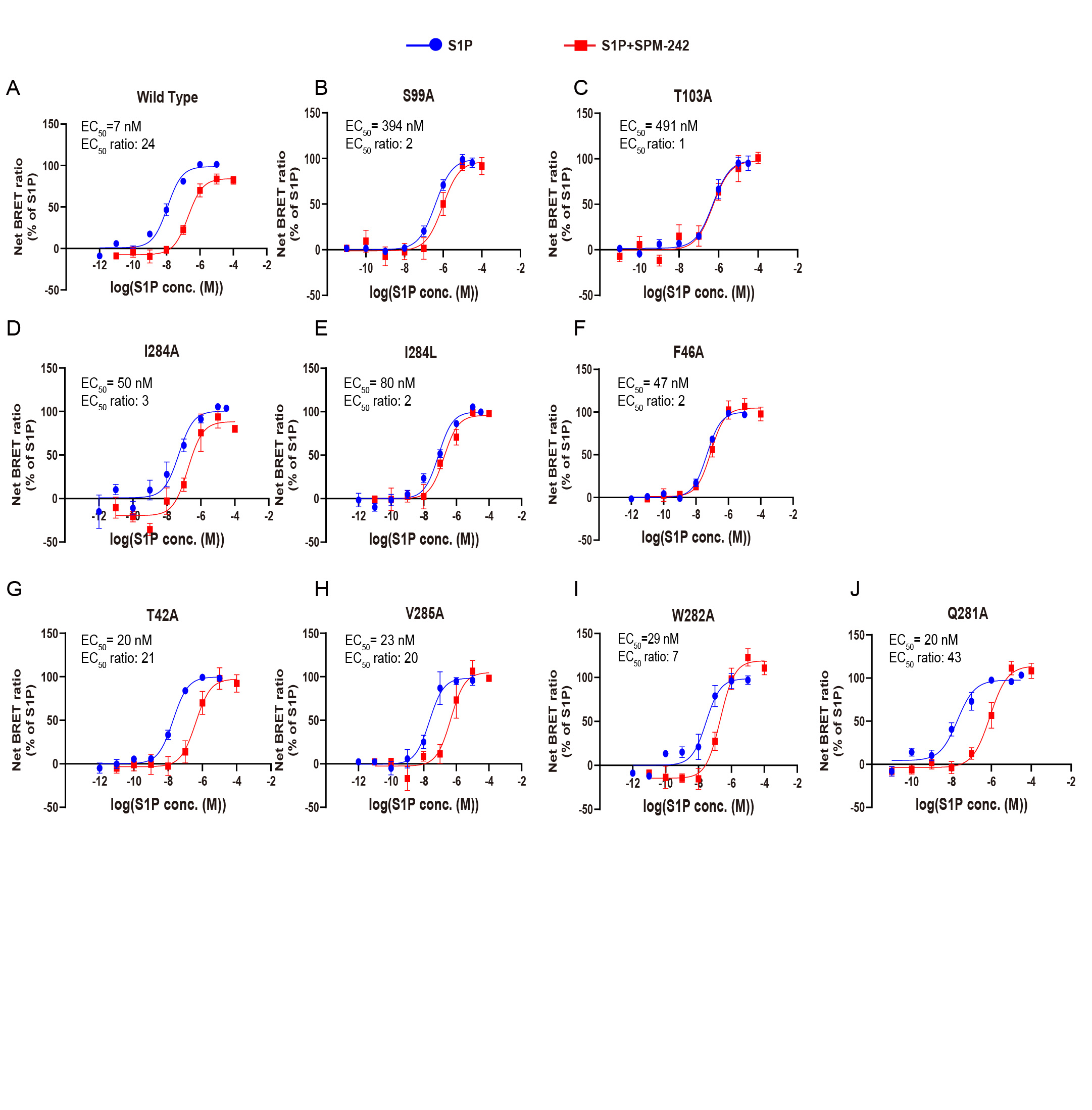


**Figure S7. TRUPATH assays of SPM-242.** TRUPATH assays of wild-type and mutant S1P_3_ induced by endogenous ligand S1P (blue) or S1P with the presence of antagonist SPM-242 (10^-7^ M, red). The assays were performed at least three independent experiments in duplicate. Data are shown as mean ± SEM. EC_50_ values of S1P and EC_50_ ratios (EC_50(S1P+SPM-242)_/EC_50(S1P)_) are shown in the top left corner for each graph. See Table S2 for detailed statistical evaluation and expression levels.

**
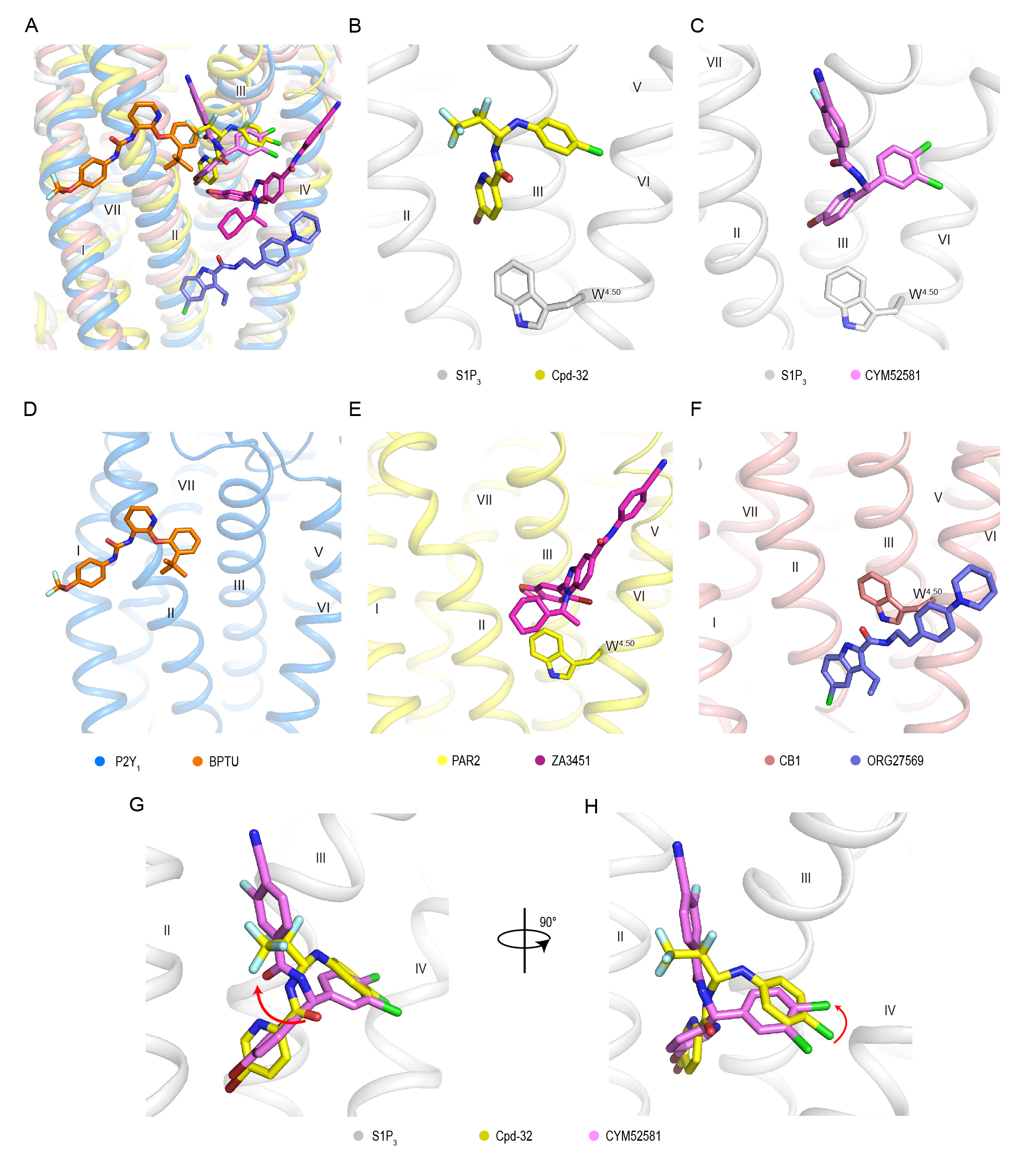
**

**Figure S8. Structural comparison of allosteric ligand-binding mode in the helices I-IV in class A GPCR.** (A) Comparison of allosteric binding-sites of S1P_3_, P2Y_1_, PAR2 and CB1. The structures of S1P_3_–SPM-242–Cpd-32, S1P_3_–SPM-242–CYM52581, P2Y_1_–BPTU (PDB code: 4XNV), PAR2–AZ3451 (PDB code: 5NDZ), and CB_1_–CP55940–ORG27569 (PDB code: 6KQI) are shown in cartoon representation, with the receptors colored grey, blue, yellow, and salmon, respectively. Their allosteric ligands are displayed as sticks and colored yellow, violet, orange, magenta, and slate, respectively. (B-F) Side view of those allosteric ligands in the corresponding receptors. W^4.50^ is chosen to as a reference. (G and H) S1P_3_ is shown in grey cartoon representation. The allosteric ligands Cpd-32 and CYM52581 are shown in sticks representation and colored yellow and violet, respectively. The red arrow indicates the rotation of carbonyl oxygen atom of acetamide moiety and *para*-chlorine atom of CYM52581 relative to Cpd-32.


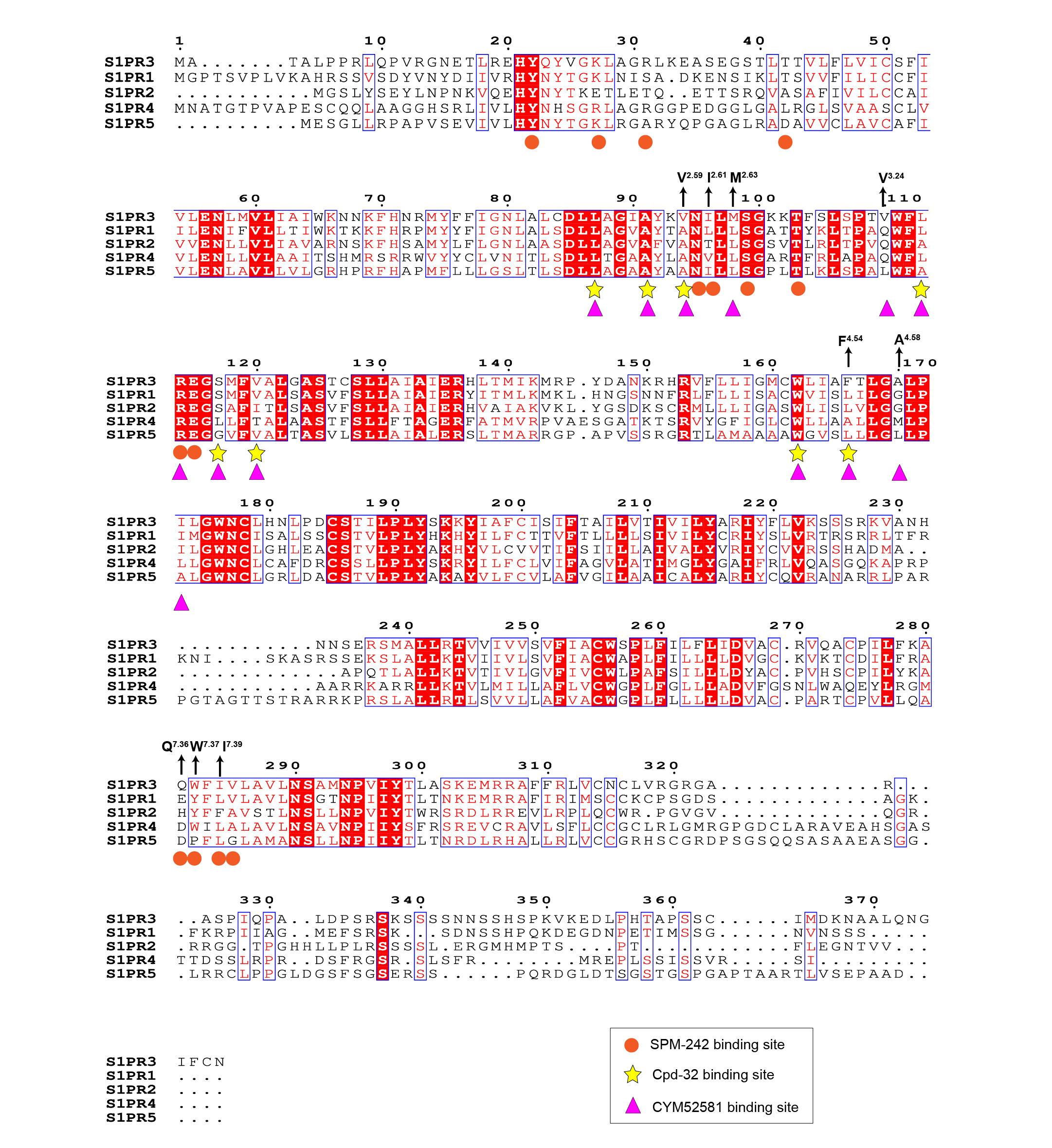


**Figure S9. Sequence alignment of the human S1P receptors.** Colors represent similarity of residues: red background, identical; red text, similar. The S1P receptors alignment was generated using UniProt (http://www.uniprot.org/align) and the graphic was prepared on the ESPrinpt 3.0 server (<https://espript.ibcp.fr/ESPript/cgi-bin/ESPript.cgi>). The black arrows indicate the key residues participated in ligands SPM-242, Cpd-32, and CYM52581variable in S1P receptors.

**Table S1. Data collection and structure refinement statics of S1P2 and S1P3 structures.**

|  | **S1P_3_–SPM-242–Cpd-32** | **S1P_3_–SPM-242–CYM52581** |
| --- | --- | --- |
| **Data collection** |  |  |
| Space group | *C222*_1_ | *C222*_1_ |
| Cell dimensions |  |  |
| *a*, *b*, *c* (Å) | 71.9, 301.7, 70.1 | 73.6, 300.0, 67.5 |
| α, β, γ（°） | 90.0, 90.0, 90.0 | 90.0, 90.0, 90.0 |
| Resolution (Å) | 38.5 - 3.0 (3.1 - 3.0)^a^ | 29.7 - 3.6 (3.7 - 3.6) |
| R*_pim_*(%) | 9.6 (36.4) | 16.7 (47.8) |
| *I*/σ*(I)* | 8.0 (1.0) | 4.4 (1.5) |
| Completeness (%) | 99.74 (98.49) | 99.53 (100.00) |
| Redundancy | 7.9 (13.5) | 7.3 (7.1) |
| **Refinement** |  |  |
| Resolution (Å) | 44.9 - 3.0 | 29.7 - 3.6 |
| No. reflections | 15,726 (1,496) | 9,019 (886) |
| *R*_work_ / *R*_free_ (%) | 0.24/0.27 | 0.23/0.28 |
| Number of atoms |  |  |
| Protein | 3,712 | 3,696 |
| Ligand | 53 | 55 |
| *B*-factors (Å^2^) |  |  |
| Protein | 76.3 | 51.4 |
| Ligand | 96.8 | 81.2 |
| R.m.s. deviations |  |  |
| Bond lengths (Å) | 0.007 | 0.004 |
| Bond angles (°) | 0.75 | 0.70 |
| Ramachandran plot (%) |  |  |
| Favored | 95.36 | 95.15 |
| Allowed | 4.64 | 4.85 |
| Disallowed | 0.00 | 0.00 |

|  | S1P_2_–G_i_ complex | S1P_3_–G_i_ complex |
| --- | --- | --- |
| **Data collection and processing** |  |  |
| Magnification | 81,000 | 81,000 |
| Voltage (kV) | 300 | 300 |
| Electron exposure (e^–^/Å^2^) | 70 | 70 |
| Defocus range (μm) | -0.8 ~ -1.5 | -0.8 ~ -1.5 |
| Pixel size (Å) | 1.045 | 1.045 |
| Symmetry imposed | C1 | C1 |
| Initial particle projections (no.) | 2,910,684 | 2,237,371 |
| Final particle projections (no.) | 415,313 | 669,267 |
| Map resolution (Å) | 3.8 | 3.0 |
| FSC threshold | 0.143 | 0.143 |
| Map resolution range (Å) | 2.5−5.0 | 2.5−5.0 |
| **Refinement** |  |  |
| Initial model used (PDB code) | 6OMM | *S1P_3_–SPM-242–Cpd-32  6OMM |
| Model resolution (Å) | 3.82 | 3.16 |
| FSC threshold | 0.5 | 0.5 |
| Map sharpening B factor (Å^2^) | −107 | -103.75 |
| Model composition |  |  |
| Non-hydrogen atoms | 6890 | 6959 |
| Protein residues | 885 (6890 atoms) | 900 (6959atoms) |
| Receptor residues | 217 (1746 atoms) | 293 (2321 atoms) |
| G protein residues | 668 (5144 atoms) | 607 (4638 atoms) |
| Antibody residues | -- | -- |
| B factors (Å^2^) |  |  |
| Protein | 68.27 | 61.52 |
| R.m.s. deviations |  |  |
| Bond lengths (Å) | 0.002 | 0.003 |
| Bond angles (°) | 0.519 | 0.557 |
| Validation |  |  |
| MolProbity score | 1.64 | 1.82 |
| Clashscore | 9.44 | 9.37 |
| Rotamer outliers (%) | 0.00 | 0.00 |
| Ramachandran plot |  |  |
| Favored (%) | 97.24 | 95.72 |
| Allowed (%) | 2.76 | 4.28 |
| Disallowed (%) | 0.00 | 0.00 |

^a^Numbers in parentheses refer to the highest-resolution shell.

**Table S2. Trupath assay of different agonists and antagonists of S1P3.**

Effect of wild-type and mutant S1P_3_ on activation S1P efficacy and antagonist SPM-242 potency using TRUPATH assay.

| **WT/mutants^a^** | **S1P** | | **S1P / SPM-242 (10^-7^ M)** | | | | **Surface expression**  **(%/ wild type)^f^** |
| --- | --- | --- | --- | --- | --- | --- | --- |
|  | **EC_50_(nM)**  **(pEC_50_ ± SEM)^b^** | **N**^c^ | **EC_50_(nM)**  **(pEC_50_ ± SEM)^b^** | **Ratio^d^** | **Ratio change^e^**  **(fold)** | **N**^c^ |  |
| WT | 8  (8.12±0.07) | 15 | 187  (6.73±0.13) | 24 | 1 | 15 | 100 |
| Y22^N^A | ND | 3 | / | / | / | / | 67±5*** |
| R31^N^A^e^ | 48  (7.32±0.08)*** | 3 | 292  (6.54±0.19) | 6 | 4 | 3 | 94±7 |
| T42^1.35^A | 20  (7.71±0.08) | 3 | 407  (6.39±0.20) | 21 | 1 | 3 | 51±3**** |
| F46^1.39^A | 47  (7.33±0.05)*** | 3 | 82  (7.08±0.12) | 2 | 14 | 3 | 91±9 |
| N95^2.60^A | ND | 3 | / | / | / | / | 59±2**** |
| I96^2.61^A | 276  (6.56±0.20)**** | 4 | 2691  (5.57±0.23)**** | 10 | 2 | 4 | 89±4 |
| I96^2.61^L | 5  (8.27±0.19) | 4 | 108  (6.97±0.18) | 20 | 1 | 4 | 87±4 |
| S99^2.64^A | 394  (6.41±0.09)**** | 4 | 951  (6.02±0.19)* | 2 | 10 | 4 | 80±6* |
| T103^ECL1^A | 491  (6.31±0.13)**** | 4 | 531  (6.28±0.20) | 1 | 22 | 4 | 78±9* |
| R114^3.28^A | ND | 3 | / | / | / | / | 77±3* |
| E115^3.29^A | ND | 3 | / | / | / | / | 97±6 |
| Q281^7.36^A | 20  (7.70±0.14) | 3 | 850  (6.07±0.13) | 43 | 0.6 | 3 | 77±2* |
| Q281^7.36^E | 9  (8.07±0.10) | 3 | 191  (6.72±0.16) | 22 | 1 | 3 | 106±4 |
| W282^7.37^A | 29  (7.54 ± 0.18)* | 3 | 211  (6.68±0.14) | 7 | 3 | 3 | 79±5* |
| W282^7.37^F | 8  (8.08±0.07) | 3 | 226  (6.65±0.15) | 27 | 1 | 3 | 84±3 |
| W282^7.37^Y | 12  (7.93±0.10) | 3 | 164  (6.78±0.12) | 14 | 2 | 3 | 73±8** |
| I284^7.39^A | 50  (7.30±0.19)*** | 3 | 176  (6.76±0.20) | 3 | 7 | 3 | 47±5**** |
| I284^7.39^L | 80  (7.09±0.11)**** | 3 | 177  (6.75±0.16) | 2 | 11 | 3 | 77±3* |
| V285^7.40^A | 23  (7.65±0.17)* | 3 | 456  (6.34±0.20) | 20 | 1 | 3 | 104±5 |

^a^All mutants were construct based on wild-type S1P_3_.

^b^Data are shown as mean ± S.E.M. from at least three independent experiments performed in technical duplicate. One-way ANOVA was performed followed by Dunnett’s post-test and compared with WT. The P value was defined as: *P<0.1;**P<0.01;***P<0.001;****P<0.0001.

^c^Sample size; the number of independent experiments performed in duplicate.

^d^The EC_50_ ratio refers to the shift between the S1P and S1P + 100 nM antagonist SPM-242 curve (EC_50(S1P + SPM-242)_/EC_50(S1P)_) and characterizes the antagonistic effect on the wild-type receptor or receptor mutants. The influences of all tested residues on antagonistic activity were determined by comparison of EC_50_ ratios between wild-type and mutant receptors. A higher ratio indicates higher antagonistic activity. A reduced EC_50_ ratio of mutant compared to the wild-type receptor was interpreted as important for the antagonist.

^e^Fold change of EC_50_ ratio refers to EC_50_ ratio (mutants)/EC_50_ ratio (wild-type). ND (not determined) indicates data which a robust concentration response curve could not be established within the concentration range tested.

^f^Protein expression level of S1P_3_ constructs at the cell surface were determined in parallel by flow cytometry with an anti-FLAG antibody (Sigma-Aldrich) and reported as percent compared to the wild-type S1P_3_ from at least three independent measurements performed in duplicate.

Antagonism effect of Cpd-32 and CYM52581 on TRUPATH assay.

| **WT/mutants^a^** | **S1P** | | | **Cpd-32** | | | **CYM52581** | | | **Surface expression**  **(%/ wild type)^f^** |
| --- | --- | --- | --- | --- | --- | --- | --- | --- | --- | --- |
|  | **EC_50_(nM)**  **(pEC_50_ ± SEM)^b^** | **Ratio**^c^  **(mut/WT)** | **N**^d^ | **IC_50_(nM)**  **(pIC_50_±SEM)^b^** | **Ratio**^e^  **(mut/WT)** | **N**^d^ | **IC_50_(nM)**  **(pIC_50_±SEM)^b^** | **Ratio**^e^  **(mut/WT)** | **n^d^** |  |
| WT | 19  (7.72±0.07) | 1 | 12 | 48  (7.31±0.06) | 1 | 11 | 20  (7.71±0.07) | 1 | 13 | 100 |
| Construct1 | 12  (7.92±0.17) | 0.6 | 3 | 119  （6.92±0.19） | 2 | 3 | 35  (7.46±0.19) | 2 | 3 | 89±1 |
| L87^2.52^A ^d^ | 13  (7.88±0.11) | 1 | 3 | 28  (7.56±0.16) | 1 | 4 | 44  (7.36±0.18) | 2 | 4 | 91±7 |
| A91^2.56^W | 57  (7.24±0.15) | 3 | 3 | 398  (6.40±0.18)*** | 8 | 4 | / | / | / | 92±2 |
| V94^2.59^A | 85  (7.07±0.18)* | 4 | 3 | 100  (7.00±0.15) | 2 | 3 | 38  (7.42±0.17) | 2 | 4 | 75±3** |
| M98^2.63^A | 16  (7.80±0.12) | 1 | 3 | / | / | / | 84  (7.08±0.19)* | 4 | 4 | 79±4* |
| M98^2.63^L | 13  (7.88±0.20) | 1 | 3 | / | / | / | 138  (6.86±0.18)** | 7 | 3 | 69±4*** |
| V110^3.24^A | 110  (6.96±0.19)** | 6 | 3 | / | / | / | 161  (6.79±0.20)*** | 8 | 3 | 72±9** |
| V110^3.24^Q | 10  (8.00±0.15) | 1 | 3 | / | / | / | 1,464  (5.83±0.20)**** | 75 | 4 | 97±6 |
| V110^3.24^L | 20  (7.69±0.12) | 1 | 3 | / | / | / | 1,106  (5.96±0.18)**** | 56 | 3 | 78±6* |
| L113^3.27^A | 8  (8.12±0.17) | 1 | 3 | 20  (7.70±0.19) | 0.4 | 4 | 96  (7.02±0.16)* | 5 | 3 | 104±8 |
| R114^3.28^A | ND | ND | 3 | / | / | / | / | / | / | 77±3* |
| S117^3.31^A | 14  (7.85±0.17) | 1 | 3 | 1,610  (5.79±0.20)**** | 33 | 4 | 952  (6.02±0.19)**** | 27 | 3 | 76±1* |
| V120^3.34^A | 13  (7.87±0.17) | 1 | 3 | 1,474  (5.83±0.19)**** | 30 | 3 | 559  (6.25±0.17)**** | 29 | 3 | 83±2 |
| V120^3.34^W | 77  (7.12±0.18)* | 4 | 5 | ND | ND | 3 | ND | ND | 3 | 72±2** |
| W162^4.50^A | ND | ND | 3 | / | / | / | / | / | / | 56±7**** |
| F166^4.54^A | 32  (7.49±0.18) | 2 | 3 | 437***  (6.36±0.17) | 9 | 4 | 81  (7.09±0.17)* | 4 | 3 | 91±5 |
| F166^4.54^L | 4  (8.38±0.18)** | 0.3 | 4 | 160  (6.80±0.21) | 3 | 3 | 56  (7.25±0.17) | 3 | 3 | 58±5**** |
| A170^4.58^L | 7  (8.17±0.12) | 0.4 | 3 | / | / | / | 47  (7.33±0.19) | 2 | 3 | 93±2 |
| A170^4.58^M | 6  (8.21±0.17) | 0.3 | 3 | / | / | / | 97  (7.01±0.16)* | 5 | 3 | 86±3 |
| A170^4.58^G | 11  (7.98±0.18) | 1 | 3 | 132  (6.88±0.12) | 3 | 3 | 24  (7.61±0.12) | 1 | 3 | 94±1 |

^a^All mutant was construct based on wild-type S1P_3_.

^b^Data are shown as mean ± S.E.M. from at least three independent experiments performed in technical duplicate. One-way ANOVA was performed followed by Dunnett’s post-test and compared with WT. The P value was defined as: *P<0.1;**P<0.01;***P<0.001;****P<0.0001.

^c^The EC_50_ ratio was shown as EC_50(mutant)_/EC_50(WT)_, indicating the shift of WT and mutant (EC_50(mutant)_/EC_50(WT)_). ND (not determined) indicates data which a robust concentration response curve could not be established within the concentration range tested.

^d^Sample size; the number of independent experiments performed in duplicate.

^e^IC_50_ for Cpd-32 and CYM52581 were determined at EC_80_ of S1P. The IC_50_ ratio refers to the shift of WT and mutant (IC_50(mutant)_/IC_50(WT)_).

^f^Protein expression level of S1P_3_ constructs at the cell surface were determined in parallel by flow cytometry with an anti-FLAG antibody and ported as percent compared with the wild-type S1P_3_ from at least three independent measurements performed in duplicate.

**Supporting Information**

**Synthesis of (–)-(*R*)-SPM-242 (4)**

(–)-(*R*)-2-Amino-4-(2-chloro-4-((3-hydroxyphenyl)thio)phenyl)-2-(hydroxy­methyl)­butyl dihydrogen phosphate (**(–)-(*R*)-SPM-242**, **(–)-(*R*)-4**) was synthesized by WuXi AppTec in analogy to the procedure described in the literature, starting from commercially available 2-amino-2-(4-((3-(benzyloxy)phenyl)thio)-2-chloro-phenethyl) propane-1,3-diol (**10**) (Scheme **2**).^[[1]](#footnote-1),^^[[2]](#footnote-2)^

**Scheme 2:** Synthesis of **(–)-(R)-SPM-242** (**(****–)-(R)-4**)

**Benzyl (4-(4-((3-(Benzyloxy)phenyl)thio)-2-chlorophenyl)-1-hydroxy-2-(hydroxymethyl)­butan-2-yl)­­carbamate (11)**

Aqueous NaHCO_3_ (2 M, 15 mL) and benzyl chloroformate (887 mg, 750 µL, 5.20 mmol) was added to a solution of 2-amino-2-(4-((3-(benzyloxy)phenyl)thio)-2-chlorophenethyl)propane-1,3-diol (**10**) (2.00 g, 4.16 mmol) in ethyl acetate (50 mL), and the mixture was stirred at room temperature for 30 min. Another portion of benzyl chloroformate (830 mg, 700 µL, 4.90 mmol) was added, and the mixture was stirred over night. Another portion of benzyl chloroformate (830 mg, 700 µL, 4.90 mmol) was added, and the mixture was stirred for 2 h. Ethyl acetate (50 mL) was added, and the phases were separated. The aqueous layer was extracted with ethyl acetate, and the combined organic layers were dried (Na_2_SO_4_) and concentrated under reduced pressure. The crude product was purified by flash column chromatography on silica (cyclohexane/ethyl acetate) to give **11** as white solid (2.12 g, 88%).

**Benzyl (4-(4-((3-(Benzyloxy)phenyl)thio)-2-chlorophenyl)-1-((bis(benzyloxy)­phosphoryl)­oxy)-2-(hydroxymethyl)butan-2-yl)carbamate (*rac*-(13))**

4-Dimethylaminopyridine (348 mg, 2.85 mmol) was added to a solution of benzyl (4-(4-((3-(benzyl­oxy)phenyl)thio)-2-chlorophenyl)-1-hydroxy-2-(hydroxymethyl)butan-2-yl)-carbamate (**11**) (1.10 g, 1.90 mmol) and dibenzyl phosphoryl chloride (**12**) (846 mg, 2.85 mmol) in dichloromethane (20 mL). The mixture was stirred at room temperature for 2 h and then concentrated under reduced pressure. The residue was purified by flash chromatography on silica (gradient cyclohexane/ethyl acetate 9:1 to cyclohexane/ethyl acetate 6:4) to give ***rac*-(13)** as colorless oil (950 mg, 59%).

**(*R*)-Benzyl (4-(4-((3-(Benzyloxy)phenyl)thio)-2-chlorophenyl)-1-((bis(benzyloxy)­phosphoryl)­oxy)-2-(hydroxymethyl)butan-2-yl)carbamate ((*R*)-(13)) and (*S*)-Benzyl (4-(4-((3-(Benzyloxy)­phenyl)­thio)-2-chlorophenyl)-1-((bis­(benzyl­oxy)­phosphoryl)oxy)-2-(hydroxymethyl)butan-2-yl)carbamate ((*S*)-(13))**

The enantiomers of ***rac*-(13)** (768 mg, 0.92 mmol) were seperated by preparative chiral HPLC (Chiralpak IC, 10 x 250 mm x 5 µM, 40% EtOH + 20 mM NH_3_ in supercritical CO_2_) to give **(*R*)-(13)** (351 mg, 45%) and **(*S*)-(13)** (371 mg, 47%), both as colorless oil.

**Early eluting enantiomer (*S*)-(13):** Retention time chiral HPLC (Chiralpak IC, 4.6 mm x 250 mm x 5 µM, 40% EtOH + 20 mM NH_3_ in supercritical CO_2_, flow 4.0 mL/min, 40 °C): 3.46 min

**Late eluting enantiomer (*R*)-(13):** Retention time chiral HPLC (Chiralpak IC, 4.6 mm x 250 mm x 5 µM, 40% EtOH + 20 mM NH_3_ in supercritical CO_2_, flow 4.0 mL/min, 40 °C): 4.14 min

**(–)-(*R*)-2-Amino-4-(2-chloro-4-((3-hydroxyphenyl)thio)phenyl)-2-(hydroxy­methyl)butyl dihydrogen phosphate ((–)-(*R*)-SPM-242, (–)-(*R*)-5)**

Iodtrimethylsilane (735 mg, 3.67 mmol) was added to a solution of (*R*)-benzyl (4-(4-((3-(benzyloxy)­phenyl)­thio)-2-chlorophenyl)-1-((bis(benzyloxy)phosphoryl)oxy)-2-(hydroxy­methyl)­butan-2-yl)carbamate (**(*R*)-(13)**) at 0 °C, and the mixture was warmed at room temperature and stirred over night. Methanol (10 mL) was added, and the mixture was concentrated under reduced pressure. The residue was purified by preparative HPLC (X-Bridge, gradient of MeCN in H_2_O, 0.1% TFA) to give **(–)-(*R*)-SPM-242 ((–)-(*R*)-5)** (117 mg, 47%) as white solid.

Optical Rotation: $\left[ \alpha\right]_{589}^{20}$= – 2.6 (c 1.00, MeOH)

S1P_3_ IC_50_: 3 nM^[[3]](#footnote-3)^

**(+)-(*S*)-2-Amino-4-(2-chloro-4-((3-hydroxyphenyl)thio)phenyl)-2-(hydroxy­methyl)­butyl dihydrogen phosphate ((+)-(*S*)-SPM-242, (+)-(*S*)-4)**

Iodtrimethylsilane (882 mg, 4.41 mmol) was added to a solution of (*S*)-benzyl (4-(4-((3-(benzyloxy)­phenyl)­thio)-2-chlorophenyl)-1-((bis(benzyloxy)phosphoryl)oxy)-2-(hydroxy­methyl)­butan-2-yl)carbamate (**(*S*)-(13)**) at 0 °C, and the mixture was warmed at room temperature and stirred overnight. Methanol (10 mL) was added, and the mixture was concentrated under reduced pressure. The residue was purified by preparative HPLC (X-Bridge, gradient of MeCN in H_2_O, 0.1% TFA) to give **(+)-(*S*)-SPM-242 ((+)-(*S*)-4)** (195 mg, 62%) as white solid.

Optical Rotation: $\left[ \alpha\right]_{589}^{20}$= +4.5 (c 1.00, MeOH)

S1P_3_ IC_50_: 44 nM^3^

**Synthesis of Cpd-32 (5)**

5‐Bromo‐*N*‐{1‐[(4‐chlorophenyl)amino]‐2,2,3,3,3‐pentafluoro­propyl}­­pyridine‐2‐carbox­amide **(Cpd-32, 5**) was synthesized by WuXi AppTec as descibed in WO11019681 (Scheme 3).^[[4]](#footnote-4)^

Scheme 3: Synthesis of **Cpd-32** (**5**)

**5-Bromo-*N*-(2,2,3,3,3-pentafluoropropyl)picolinamide (15)**

A mixture of 5-bromopyridine-2-carboxamide (**14**, 2.00g, 9.50 mmol) and pentafluorpropionaldehyde hydrate (3.30g, 19.90 mmol) in THF (20 mL) is heated at 80 °C for 4 h. All volatiles are removed under reduced pressure, and the residue is purified by flash chromatography on silica (cyclohexane/ethyl acetate 8:2) to give the title compound **15** (2.44g, 70%) as white solid.

**5‐Bromo‐*N*‐{1‐[(4‐chlorophenyl)amino]‐2,2,3,3,3‐pentafluoro­propyl}­­pyridine‐2‐carbox­amide (Cpd-32, 5)**

A mixture of 5-bromo-*N*-(2,2,3,3,3-pentafluoropropyl)picolinamide (**15**, 300 mg, 0.86 mmol) in chloroform (5.0 mL) was treated with phosphorous pentachloride (178mg, 0.86 mmol) and stirred at 70 °C for 48 h. The mixture is cooled at room temperature, and water (2 mL) is added. The phases are separated and the organic layer is treated with para-chloroaniline (219 mg, 1.72 mmol) and stirred at room temperature over night. All volatiles are removed under reduced pressure, and the residue is purified by prepartive reversed-phase HPLC (X-Bridge, gradient of MeCN in H_2_O, 0.1% NH_3_) to give **Cpd-32** (**5**) (50 mg, 12%) as white solid.

S1P_3_ IC_50_: 3 nM^3^

**Synthesis of (–)-(*R*)-CYM52581 ((–)-(*R*)-6)**

((*R*)-*N*‐[(5‐bromopyridin‐2‐yl)(3,4‐dichlorophenyl)methyl]‐4‐cyano‐3‐fluoro­benz­amide) **((*R*)-CYM52581, (*R*)-6**) was synthesized by WuXi AppTec as described in WO16053855 using (*R*)-*tert*-butylsulfinamide (Scheme 2).^[[5]](#footnote-5)^

Scheme 2: Synthesis of **(–)-(*R*)-CYM52581** (**(–)-(*R*)-6**)

**(*R*)-*N*-((5-bromopyridin-2-yl)methylene)-2-methylpropane-2-sulfinamide (17)**

A mixture of (*R*)-(+)-2-methyl-2-propanesulfinamide (0.80 g, 6.45 mmol), 5‑bromo­pyridine-2-carbaldehyde (**16**, 1.00 g, 5.38 mmol) and titanium ethoxide (4.00 g, 17.54 mmol) was heated at 70 °C for 1 h. Ethyl acetate and brine were added, and the mixture was filtered. The phases were separated and the aqueous phase was extracted with ethyl acetate. The combined organic layers were dried (Na_2_SO_4_) and concentrated under reduced pressure to give **17** (1.58 g, 99%) as yellow solid, which was directly used in the next step without further purification.

**(*R*)-*N*-((*R*)-(5-Bromopyridin-2-yl)(3,4-dichlorophenyl)methyl)-2-methylpropane-2-sulfinamide (18)**

(3,4-Dichlorophenyl)magnesium chloride (0.5 M in THF, 30.0 mL, 15.0 mmol) was added at dropwise to a precooled solution of (*R*)-*N*-((5-bromopyridin-2-yl)methylene)-2-methylpropane-2-sulfinamide (**17**, 1.60 g, 5.33 mmol) in dry THF (30 mL) at -78 °C, and the mixture was stirred at -78°C for 1 h. Saturated aqueous NH_4_Cl was added, and the mixture was allowed to warm to room temperature and extracted twice with ethyl acetate. The combined organic layers were dried over Na_2_SO_4_ and concentrated under reduced pressure. The residue was purified by flash chromatography on silica (gradient cyclohexane to cyclohexane/ethyl acetate 40:60) to give **18** (1.80 g, 71%) as pale yellow oil.

**(*R*)-(5-Bromopyridin-2-yl)(3,4-dichlorophenyl)methanamine hydrochloride (19*HCl)**

(*R*)-*N*-((*R*)-(5-Bromopyridin-2-yl)(3,4-dichlorophenyl)methyl)-2-methylpropane-2-sulfinamide (**18**) (350 mg, 0.80 mmol) was added to a solution of hydrogen chloride in ethyl acetate (4 M, 20 ml, 80 mmol) at 15 °C, and the mixture was stirred for 1 h. The mixture was filtered, and the residue was washed with ethyl acetate and dried to give

(*R*)-(5-bromopyridin-2-yl)(3,4-dichlorophenyl)methanamine hydrochloride (**19*HCl**, 250 mg, 85%) as white solid.

**(–)-(*R*)-*N*-((5-Bromopyridin-2-yl)(3,4-dichlorophenyl)methyl)-4-cyano-3-fluoro­benz­amide** (**(–)-(*R*)-CYM52581**, **(–)-(*R*)-6**)

1-Hydroxybenzotriazole (48 mg, 0.36 mmol), 1-(3-dimethylaminopropyl)-3-ethyl­carbo­diimid (69 mg, 0.45 mmol) and *N*,*N*‑diisopropylethylamine (136 mg, 101 µL, 1.05 mmol) was added to a solution of (*R*)-(5-bromopyridin-2-yl)(3,4-dichloro­phenyl)­methanamine hydrochloride (**19*HCl**, 111 mg) in dichloromethane (1.0 ml). 4-Cyano-3-fluorobenzoic acid (60 mg, 0.36 mmol) was added, and the reaction was stirred for 2 h. Dichloromethane was added, and the mixture was washed with water and brine. The mixture was dried (Na_2_SO_4_) and concentrated under reduced pressure. The residue was purified by flash chromatography on silica (gradient cyclohexane/ethyl acetate 20:1 to cyclohexane/ethyl acetate 2:1) to give **(–)-(*R*)-CYM52581** (**(–)-(*R*)-6**), 57 mg, 35%) as white solid.

Optical rotation: $\left[ \alpha\right]_{589}^{20}$= – 2.8 (c 1.00, DMSO)

S1P_3_ IC_50_: 6 nM^3^

1. Jo, E. et al. Novel selective allosteric and bitopic ligands for the S1P(3) receptor. *ACS Chem Biol* **7**, 1975-1983 (2012). [↑](#footnote-ref-1)
2. Kohno, Y., Tanaka, K., Kuriyama, K., Hori, W., Aminophosphonic Acid Derivatives, Addition Salts thereof and S1P Receptor Modulators, WO 2004/074297, 2004 [↑](#footnote-ref-2)
3. Potencies of S1P3 inhibitors were determined in CHO-K1 cells stably expressing the human S1P3 receptor via detection of intracellular cAMP content. Inhibition of S1P-stimulated cAMP decrease was measured using Cisbio HTRF technology. [↑](#footnote-ref-3)
4. Nguyen, P. X., Heidelbaugh, T. M., Chow, K., Garst, M. E, Selective Sphingosine-1-phosphate Receptor Antagonists, WO2011/019681, 2011. [↑](#footnote-ref-4)
5. Roberts, E., Rosen, H., Urbano, M., Guerrereo, M., Sphingosine-1-phosphate Receptor Modulators for Treatment of Cardiopulmonary Disorders, WO 2016/053855, 2016. [↑](#footnote-ref-5)
